# q-rung orthopair fuzzy 2-tuple linguistic clustering algorithm and its applications to clustering analysis

**DOI:** 10.1038/s41598-023-29932-y

**Published:** 2023-02-16

**Authors:** Fatima Abbas, Jawad Ali, Wali Khan Mashwani, Muhammad I. Syam

**Affiliations:** 1 Institute of Numerical Sciences, Gomal University, D. I. Khan, KPK Pakistan; 2 Institute of Numerical Sciences, Kohat University of Science and Technology, Kohat, KPK Pakistan; 3 Department of Mathematical Sciences, United Arab Emirates University, P. O. Box 15551 Al-Ain, UAE

**Keywords:** Engineering, Mathematics and computing

## Abstract

q-ROPFLS, including numeric and linguistic data, has a wide range of applications in handling uncertain information. This article aims to investigate q-ROPFL correlation coefficient based on the proposed information energy and covariance formulas. Moreover, considering that different q-ROPFL elements may have varying criteria weights, the weighted correlation coefficient is further explored. Some desirable characteristics of the presented correlation coefficients are also discussed and proven. In addition, some theoretical development is provided, including the concept of composition matrix, correlation matrix, and equivalent correlation matrix via the proposed correlation coefficients. Then, a clustering algorithm is expanded where data is expressed in q-ROPFL form with unknown weight information and is explained through an illustrative example. Besides, detailed parameter analysis and comparative study are performed with the existing approaches to reveal the effectiveness of the framed algorithm.

## Introduction

MCGDM problem is one of the most significant everyday activities^1–6^. At first, the assessment information is often represented in terms of real numbers. In many complicated MCGDM scenarios, however, it is preferable to ascribe values using fuzzy numbers or linguistic phrases due to the uncertainty and ambiguity of human comprehension. Therefore, Zadeh^7^ concocted the LVR to characterize the qualitative data in decision-making situations. LVRs may cope with situations when it is difficult to quantify the evaluation values of criteria. For instance, a student’s examination results may be represented exactly using numerical numbers, yet his or her morals are often assessed using verbal phrases such as “bad, fair, and good.” Moreover, Herrera and Martinez^8^ suggested a model of 2TL representation composed of a linguistic term and a real number in the range $$[- 0.5, 0.5)$$. The framework can exemplify any computing results throughout the aggregation procedure, so it successfully prevents information loss. Various studies on the scope of 2-tuple linguistic terms^9–12^ have been done in recent years. These additions enable the efficient expression of ill-defined and fuzzy data while solving the MCGDM issue. Later, Wei et al.^13^ expanded the 2TL model to the P2TLN, which may convey a linguistic term’s membership grade $$\mu$$ and non-membership grade $$\nu$$. Some conventional MCGM approaches, such as Taxonomy method^14^, VIKOR method^14^, and TODIM method^15^, are expanded to Pythagorean 2TL information based on this concept. However, the assignment of membership grades and non-membership grades in P2TLNs is subject to certain constraints. P2TLNs have a requirement that $$\mu ^2+\nu ^2\le 1$$, yet there are several instances in which the assessment information given by DMs in the form of P2TLNs cannot satisfy the requirement. For instance, if the membership and non-membership grades are given as $$\left\langle 0.7, 0.8\right\rangle$$, P2TLNs are unable to successfully process it because $$0.7^2+0.8^2>1$$. Wei et al.^16^ brought forward the q-ROPFLSs based on the q-rung orthopair fuzzy sets^17^, in which the total of the qth power of membership grade and non-membership grade should be less than 1, i.e., $$\mu ^q+\nu ^q\le 1$$. And when $$q=2$$, the q-ROPFLN can be reduced to P2TLN. The q-ROPFLN is hence a more generic and adaptable type of information representation. It has an exceptionally vast expression domain and can prevent data loss. Wei et al.^16^ presented various q-ROPFL Heronian mean operators as well as their weighted versions. Ju et al.^18^ investigated some Muirhead mean operators with q-ROPFLNs for MCGDM challenges. Recently, Li et al.^19^ devised q-ROPFL PROMETHEE II model for MCGDM with unknown weight information.

In recent decades, the correlation coefficient, which is a key for studying the link between any two parameters or variables, has garnered considerable attention. The correlation coefficient developed by Karl Pearsons^20^ has been utilized in several statistical research, including data analysis and classification, pattern identification, clustering, medical diagnosis, and decision-making. It has been determined that conventional correlation is unsuitable for handling data pertaining to situations of a fuzzy character. To address such issues, several writers have expanded the concept of statistical correlation to include fuzzy correlation^21–23^. In^24^, Gerstenkon and Manko developed the notion of the intuitionistic fuzzy correlation coefficient. Hong and Hwang^25^ analyzed the correlation measure and correlation coefficient for IFSs in probabilistic spaces. Zeng and Li^26^ presented the correlation coefficient of IFSs, which is analogous to the cosine of the intersectional angle in finite sets and probability spaces. Another study^27^ demonstrated the applicability of IFS correlation coefficients to pattern recognition issues. Chen et al.^28^ developed correlation coefficients for hesitant fuzzy sets and used these concepts to clustering analysis. The authors in^29^ explored multilevel analysis methodologies and applications. Garg^30^ developed a novel correlation coefficient for Pythagorean fuzzy sets and used them for decision making. Park et al.^31^ propounded the correlation coefficient of interval-valued IFSs and highlighted their applicability by applying them to the challenges of MCGDM. Nguyen^32^ devised the similarity or dissimilarity measure for IFSs with its applications in pattern recognition, whereas Du^33^ produced the correlation and correlation coefficients of q-ROFSs. Recently, Li and his coworkers^34^ studied two $\zeta$-correlation coefficients for q-rung orthopair fuzzy setting and addressed an example of clustering analysis to justify the superiority of the suggested approach.

The advancement of the theory and the practical uses of correlation coefficients motivated us to investigate these concepts. Following that, this article explores the correlation coefficients and clustering technique for q-ROPFLSs. Unlike the aforesaid fuzzy sets, q-ROPFLS is constituted by a linguistic 2-tuple and a q-ROFS. Concerning the linguistic 2-tuple, it is a model that prevents the loss of information during computations of discrete linguistic values. The intuitionistic 2TL sets and the Pythagorean 2TL sets are likewise composed of a linguistic 2-tuple, but they include limits on the selection of membership and non-membership grades, whereas q-ROPFLS do not. In the context of intuitionistic 2TL sets, we cannot award 0.5 and 0.6 as membership and non-membership grades since their total exceeds 1. Similarly, in the context of Pythagorean 2TL sets, we cannot select 0.7 and 0.8 as membership and non-membership grades due to the limitation that the sum of their squares exceeds 1. In q-ROFS, however, the range of numbers that can be allocated as membership and non-membership grades is so broad, i.e., we can assign membership and non-membership grades any value between 0 and 1. Thus, the structure of q-ROPFLS is superior compared to other existing frameworks.

The factors listed inspired us to perform this study:

(I) The q-ROPFLS is a useful tool for conveying MCGM problem assessment information. The type of information itself reveals that it mainly contains the following key advantages: (i) information distortion during linguistic information processing can be reduced; (ii) information loss through incorporating parameter q to convey evaluation results can be effectively avoided, and a significantly large range can be used to represent membership grades and non-membership grades for a linguistic evaluation; and (iii) the problems associated with two-dimensional information can be effectively addressed in real-world applications. Currently, there are several techniques in the literature that work in information energy and develop a formula for calculating the correlation coefficient; but, there is a need to expand the methodology for measuring the correlation coefficient in the context of q-ROPFLS.
(II) The significance of criteria in decision analysis is of the utmost relevance for making rational choices. Typically, these weights are unavailable in advance. However, the majority of available clustering methods only account for the situation of known weights and disregard the case of unknown weights. To get more precise findings, it is required to develop a clustering model based on unknown criteria weights.

According to the aforementioned motivations, the following are the novel aspects of this research study:

(I) Information energy, covariance, correlation coefficient, and their corresponding weighted forms for q-ROPFTLSs are formulated. Also, the required properties of the proffered formulation are verified.
(II) Based on the developed theory, the conventional clustering algorithm is extended for q-ROPFLSs with unknown criteria weight information.
(III) A case concerning the classification of CIM software is provided to demonstrate the application of the framed algorithm. Then, a case concerning the clustering of construction materials is studied to compare the proposed method with the prevailing methods.

The remaining sections of the paper are prepared as follows. In Section “Preliminaries”, a brief introduction to fundamental ideas is supplied. Section “Correlation coefficient for q-ROPFLSs” gives the notion of q-ROPFL correlation coefficient and weighted correlation coefficient along with their important properties. In Section “Clustering algorithm under q-ROPFL environment”, clustering algorithm is described to handle the clustering problems with q-ROPFL data. Section “Applications and analysis” analyze the presented algorithm via examples, experiments, and comparisons. At last, Section “Concluding remarks” concludes the article.

**Figure 1:**
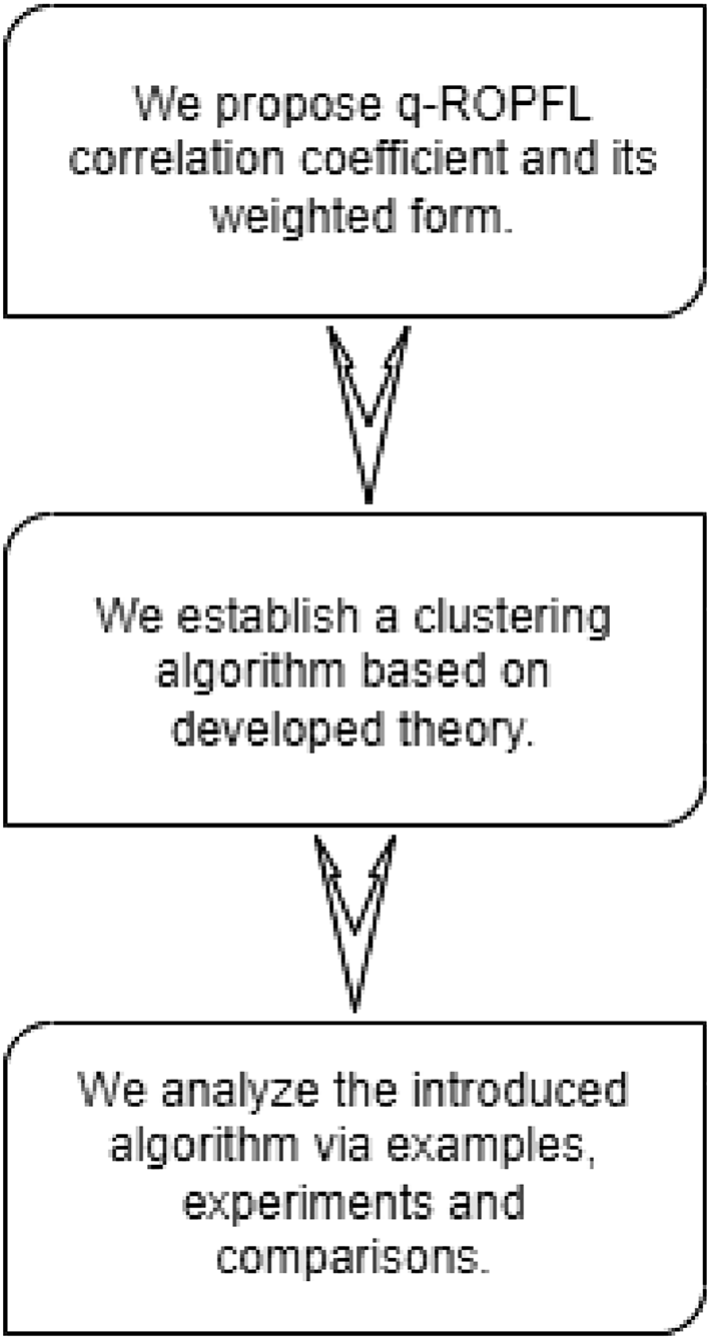
Graphical illustration of the proposed work.

The main theme of this study is visualized in Fig. 1.

## Preliminaries

In what follows, we make a brief review of LTS, IFLS and q-ROPFLS.

Let $S=\left\{ s_{\theta }|\theta =1,2,...,\ell \right\}$ be a linguistic term set with odd cardinality. Any label, $s_{\theta }$ indicates a possible value for a linguistic variable, and it must meet the following requirements^8,35^:

1. Ordered set: $${{s_{\theta }}_1}\le {{s_{\theta }}_2} \Leftrightarrow \theta _1\le \theta _2;$$
2. Negation operator: $Neg\left( {{s_{\theta }}_1} \right) ={{s_{\theta }}_2}$, such that $\theta _1+ \theta _2=\ell ;$
3. Max operator: $\max \left( {{s_{\theta }}_1},{{s_{\theta }}_2}\right) ={{s_{\theta }}_1}$ if ${{s_{\theta }}_1}\ge {{s_{\theta }}_2};$
4. Min operator: Min operator: $\min \left( {{s_{\theta }}_1},{{s_{\theta }}_2}\right) ={{s_{\theta }}_1}$ if ${{s_{\theta }}_1}\le {{s_{\theta }}_2}.$

For instance, *S* can be defined as

$$\begin{aligned} S=\left\{ \begin{array}{c} s_1 = \text {extremely poor}, s_2 = \text {very poor}, s_3 = \text {poor}, s_4 = \text {medium}, s_5 = \text {good}, s_6 = \text {very good}, \\ s_7 = \text {extremely good} \end{array}\right\} . \end{aligned}$$

Based on the notion of symbolic translation, Herrera and Martinez^8,35^ created the 2-tuple fuzzy linguistic representation model. It is used to express linguistic evaluation information as a 2-tuple $\left( s_{\theta },\varkappa _{} \right)$ where $s_{\theta }$ is a linguistic label from the predefined linguistic term set *S*, $\varkappa _{}$ is the value of symbolic translation, and $\varkappa _{}\in [-0.5, 0.5)$.

### Definition 1

^8,35^ Let $\vartheta$ be the result of an aggregation of the indices of a set of labels evaluated in a linguistic term set *S*, i.e., the outcome of a symbolic aggregation operation, $\vartheta \in \left[ 1,\ell \right]$, with $\ell$ being the cardinality of *S*. If $r = round(\vartheta )$ and $\varkappa _{}=\vartheta -r$ are two numbers such that $r\in \left[ 1,\ell \right]$ and $\varkappa _{} \in [-0.5, 0.5)$, then $\varkappa _{}$ is called a symbolic translation.

### Definition 2

^8,35^ Let $S=\left\{ s_{\theta }|\theta =1,2,...,\ell \right\}$ be a linguistic term set and $\vartheta \in \left[ 1,\ell \right]$ be a numerical value indicating the linguistic symbolic aggregation outcome. Then, the function $\curlywedge$ used to retrieve the 2-tuple linguistic information equivalent to $\vartheta$ is then defined as

1 $$\begin{aligned} \curlywedge : \left[ 1, \ell \right] \longrightarrow S \times [-0.5, 0.5) \end{aligned}$$

2 $$\begin{aligned} \curlywedge \left( \vartheta \right) ={\left\{ \begin{array}{ll} s_r, &{} r=round\left( \vartheta \right) \\ \varkappa _{}=\vartheta -r, &{} \varkappa _{} \in [-0.5, 0.5). \end{array}\right. } \end{aligned}$$

where round $\left( .\right)$ is the conventional round function, $s_r$ is the index label closest to $\vartheta$, and $\varkappa _{}$ is the symbolic translation value.

### Definition 3

^8,35^ Let $S=\left\{ s_{\theta }|\theta =1,2,...,\ell \right\}$ be a linguistic term set and $\left( s_r, \varkappa \right)$ be a 2-tuple. There is always a function $\curlyvee$ can be defined, such that, from a 2-tuple $\left( s_r, \varkappa \right)$ it return its equivalent numerical value $\vartheta \in \left[ 1, \ell \right]$, which is

3 $$\begin{aligned} \curlyvee : S \times [-0.5, 0.5) \longrightarrow \left[ 1, \ell \right] \end{aligned}$$

4 $$\begin{aligned} \curlyvee \left( s_r,\varkappa \right) =r+\varkappa =\vartheta . \end{aligned}$$

### Definition 4

^16^ A q-ROPFLS ${\mathcal {F}}$ on a fixed set $\texttt {Z}$ is defined as

5 $$\begin{aligned} {\mathcal {F}}=\left\{ \left( \left( s_{r(t_i)},\varkappa (t_i)\right) ,\left\langle \mu (t_i), \nu (t_i)\right\rangle \right) \right\} , \end{aligned}$$

where $s_{r(t_i)} \in S$, $\varkappa (t_i) \in [-0.5,0.5)$, $\mu (t_i),\nu (t_i) \in [0,1]$, with the condition $0\le \mu ^q(t_i)+\nu ^q(t_i)\le 1$ $\left( q\ge 1 \right)$ $\forall \; t_i\in \texttt {Z}$. The numbers $\mu (t_i),\nu (t_i)$ present the grade of membership and grade of nonmembership of the element $t_i$ to linguistic variable $\left( s_{r(t_i)},\varkappa (t_i)\right)$, respectively. Moreover, $\pi _{{\mathcal {F}}}\left( t_i \right) =1-\left( \mu ^q(t_i)+\nu ^q(t_i) \right) ^{1/q}$ is called refusal grade, and the q-rung orthopair fuzzy 2-tuple linguistic number (q-ROPFLN) is symbolized by $\partial =\left( \left( s_{r},\varkappa \right) ,\left\langle \mu , \nu \right\rangle \right) .$

### Definition 5

^16^ Let $\partial _1=\left( \left( s_{{r}_1},{\varkappa }_1\right) ,\left\langle {\mu }_1, {\nu }_1\right\rangle \right)$ and $\partial _2=\left( \left( s_{{r}_2},{\varkappa }_2\right) ,\left\langle {\mu }_2, {\nu }_2\right\rangle \right)$ be two q-ROPFLNs. Then their basic operational laws are given as follows:

1. $$\partial _1\oplus \partial _2=\left( \curlywedge \left( \curlyvee \left( s_{{r}_1},{\varkappa }_1\right) +\curlyvee \left( s_{{r}_2},{\varkappa }_2\right) \right) , \left\langle \left( {\mu }^q_1+{\mu }^q_2-{\mu }^q_1{\mu }^q_2\right) ^{1/q},{\nu }_1{\nu }_2\right\rangle \right) ;$$
2. $$\partial _1\otimes \partial _2=\left( \curlywedge \left( \curlyvee \left( s_{{r}_1},{\varkappa }_1\right) \cdot \curlyvee \left( s_{{r}_2},{\varkappa }_2\right) \right) , \left\langle {\mu }_1{\mu }_2,\left( {\nu }^q_1+{\nu }^q_2-{\nu }^q_1{\nu }^q_2\right) ^{1/q}\right\rangle \right) ;$$
3. $$\eta \partial _1=\left( \curlywedge \left( \eta \curlyvee \left( s_{{r}_1},{\varkappa }_1\right) \right) ,\left\langle \left( 1-\left( 1-{\mu }^q_1\right) ^\eta \right) ^{1/q},{\nu }^{\eta }_1 \right\rangle \right) \; \; \eta > 0;$$
4. $$\partial ^{\eta }_1=\left( \curlywedge \left( \left( \curlyvee \left( s_{{r}_1},{\varkappa }_1\right) \right) ^{\eta }\right) ,\left\langle {\mu }^{\eta }_1,\left( 1-\left( 1-{\nu }^q_1\right) ^\eta \right) ^{1/q}\right\rangle \right) \; \; \eta > 0.$$

### Definition 6

^18^ Let $\partial _{\iota }= \left( \left( s_{{r}_i},{\varkappa }_i\right) ,\left\langle {\mu }_i, {\nu }_i\right\rangle \right) \left( i=1,2,...,m \right)$ be a family of q-ROPFLNs, then q-ROPFLWA operator is:

6 $$\begin{aligned}{} & {} QROPFLWA \left( \partial _1,\partial _2,...,\partial _m \right) =\oplus ^{m}_{i=1}w_{i}\partial _{i}\nonumber \\{} & {} \quad =\left( \curlywedge \left( \sum _{i=1}^{m}w_{i}\curlyvee \left( s_{{r}_i},{\varkappa }_i\right) \right) , \left\langle \left( 1-\prod _{i=1}^{m}\left( 1- {\mu }^q_i \right) ^{w_i}\right) ^{1/q}, \prod _{i=1}^{m}\nu _i^{w_{i}}\right\rangle \right) \end{aligned}$$

where $w=\left( w_1,w_2,...,w_\flat \right) ^T$ is the weight vector of $\partial _i\left( \iota =1,2,...,m \right)$ such that $w_{i}>0$ and $\sum _{i=1}^{m}w_i=1.$ Especially, if $w=\left( \frac{1}{\flat },\frac{1}{\flat },...,\frac{1}{\flat } \right) ^T$, then the q-ROPFLWA operator reduces to q-ROPFLA operator of dimension *m*, which is described as follows:

7 $$\begin{aligned}{} & {} QROPFLA \left( \partial _1,\partial _2,...,\partial _m \right) =\frac{1}{m}\oplus ^{m}_{i=1}\partial _{i}\nonumber \\{} & {} \quad =\left( \curlywedge \left( \frac{1}{m}\sum _{i=1}^{m}\curlyvee \left( s_{{r}_i},{\varkappa }_i\right) \right) , \left\langle \left( 1-\prod _{i=1}^{m}\left( 1- {\mu }^q_i \right) ^{\frac{1}{m}}\right) ^{1/q}, \prod _{i=1}^{m}\nu _i^{\frac{1}{m}}\right\rangle \right) . \end{aligned}$$

### Definition 7

^19^ Let $\partial _1=\left( \left( s_{{r}_1},{\varkappa }_1\right) ,\left\langle {\mu }_1, {\nu }_1\right\rangle \right)$ and $\partial _2=\left( \left( s_{{r}_2},{\varkappa }_2\right) ,\left\langle {\mu }_2, {\nu }_2\right\rangle \right)$ be two q-ROPFLNs. Then the Hamming distance between $\partial _1$ and $\partial _2$ is given by

8 $$\begin{aligned} d\left( \partial _1,\partial _2 \right) =\frac{1}{2 \ell }\left[ \left| \left( 1+{\mu }^q_1-{\nu }^q_1 \right) .\curlyvee \left( s_{r_1},{\varkappa }_1 \right) - \left( 1+{\mu }^q_2-{\nu }^q_2 \right) .\curlyvee \left( s_{r_2},{\varkappa }_2 \right) \right| \right] . \end{aligned}$$

## Correlation coefficient for q-ROPFLSs

### Covariance and correlation coefficient of q-ROPFLSs

#### Definition 8

Let $\texttt {Z}$ be a fixed set, $S=\left\{ s_{\theta }|\theta =1,2,...,\ell \right\}$ be a linguistic term set, for q-ROPFLS ${\mathcal {F}}=\left\{ \left( \left( s_{r(t_i)},\varkappa (t_i)\right) ,\left\langle \mu (t_i), \nu (t_i)\right\rangle \right) \right\}$ on $\texttt {Z}$, the information energy is given by:

9 $$\begin{aligned} \textit{E}\left( {\mathcal {F}}\right) =\frac{1}{n}\sum _{i=1}^{n}\left( \left( \frac{\curlyvee \left( s_{r(t_i)},\varkappa (t_i)\right) }{\ell } \right) ^2\mu (t_i)^{2q}+\left( \frac{\curlyvee \left( s_{r(t_i)},\varkappa (t_i)\right) }{\ell } \right) ^2\nu (t_i)^{2q}\right) \end{aligned}$$

The suggested information energy meets the fuzzy interval requirement, i.e., is $0\le \textit{E}\left( {\mathcal {F}}\right) \le 1$.

#### Definition 9

Let $\texttt {Z}$ be a fixed set, $S=\left\{ s_{\theta }|\theta =1,2,...,\ell \right\}$ be a linguistic term set, for q-ROPFLSs ${\mathcal {F}}=\left\{ \left( \left( s_{r(t_i)},\varkappa (t_i)\right) ,\left\langle \mu (t_i), \nu (t_i)\right\rangle \right) \right\}$ and $\breve{{\mathcal {F}}}=\left\{ \left( \left( s_{\breve{r}(t_i)},\breve{\varkappa }(t_i)\right) ,\left\langle \breve{\mu }(t_i), \breve{\nu }(t_i)\right\rangle \right) \right\}$ on $\texttt {Z}$, the covariance of ${\mathcal {F}}$ and $\breve{{\mathcal {F}}}$ is presented by the following formula:

10 $$\begin{aligned}{} & {} \textit{K}\left( {\mathcal {F}},\breve{{\mathcal {F}}}\right) =\frac{1}{n}\sum _{i=1}^{n} \left[ \frac{1}{\ell ^2}\left\{ \begin{array}{c} \left( \curlyvee \left( s_{r(t_i)},\varkappa (t_i)\right) \cdot \curlyvee \left( s_{\breve{r(t_i)}}, \breve{\varkappa }(t_i)\right) \cdot \mu (t_i)^{q}\cdot \breve{\mu (t_i)^{q}}\right) + \\ \left( \curlyvee \left( s_{r(t_i)},\varkappa (t_i)\right) \cdot \curlyvee \left( s_{\breve{r}(t_i)}, \breve{\varkappa }(t_i)\right) \cdot \nu (t_i)^{q}\cdot \breve{\nu (t_i)^{q}}\right) \end{array}\right\} \right] \nonumber \\{} & {} \quad =\frac{1}{n}\sum _{i=1}^{n}\left[ \frac{1}{\ell ^2}\left( \curlyvee \left( s_{r(t_i)}, \varkappa (t_i)\right) \cdot \curlyvee \left( s_{\breve{r(t_i)}},\breve{\varkappa }(t_i)\right) \right) \left\{ \left( \mu (t_i)^{q}\cdot \breve{\mu (t_i)^{q}}\right) +\left( \nu (t_i)^{q}\cdot \breve{\nu (t_i)^{q}}\right) \right\} \right] \end{aligned}$$

#### Theorem 1

The covariance of two q-ROPFLSs ${\mathcal {F}}$ and $\breve{{\mathcal {F}}}$ holds the following properties:

1. $$\textit{K}\left( {\mathcal {F}},{\mathcal {F}}\right) =\textit{E}\left( {\mathcal {F}}\right) ;$$
2. $$\textit{K}\left( {\mathcal {F}},\breve{{\mathcal {F}}}\right) =\textit{K}\left( \breve{{\mathcal {F}}},{\mathcal {F}}\right)$$.

#### Proof

1. From Eq. (10), the covariance of ${\mathcal {F}}$ with ${\mathcal {F}}$ is given as

$$\begin{aligned}{} & {} \textit{K}\left( {\mathcal {F}},{\mathcal {F}}\right) =\frac{1}{n}\sum _{i=1}^{n} \left[ \frac{1}{\ell ^2}\left( \curlyvee \left( s_{r(t_i)}, \varkappa (t_i)\right) \cdot \curlyvee \left( s_{r(t_i)},\varkappa (t_i)\right) \right) \left\{ \left( \mu (t_i)^{q}\cdot \mu (t_i)^{q}\right) +\left( \nu (t_i)^{q}\cdot \nu (t_i)^{q}\right) \right\} \right] \\{} & {} \quad =\frac{1}{n}\sum _{i=1}^{n}\left( \left( \frac{\curlyvee \left( s_{r(t_i)},\varkappa (t_i)\right) }{\ell } \right) ^2\mu (t_i)^{2q}+\left( \frac{\curlyvee \left( s_{r(t_i)},\varkappa (t_i)\right) }{\ell } \right) ^2\nu (t_i)^{2q}\right) =\textit{E}\left( {\mathcal {F}}\right) . \end{aligned}$$

2. By using Eq. (10), the covariance of ${\mathcal {F}}$ and $\breve{{\mathcal {F}}}$ is given as

$$\begin{aligned}{} & {} \textit{K}\left( {\mathcal {F}},\breve{{\mathcal {F}}}\right) =\frac{1}{n}\sum _{i=1} ^{n}\left[ \frac{1}{\ell ^2}\left( \curlyvee \left( s_{r(t_i)}, \varkappa (t_i)\right) \cdot \curlyvee \left( s_{\breve{r(t_i)}},\breve{\varkappa }(t_i)\right) \right) \left\{ \left( \mu (t_i)^{q}\cdot \breve{\mu (t_i)^{q}}\right) +\left( \nu (t_i)^{q}\cdot \breve{\nu (t_i)^{q}}\right) \right\} \right] \\{} & {} \quad =\frac{1}{n}\sum _{i=1}^{n}\left[ \frac{1}{\ell ^2}\left( \curlyvee \left( s_{\breve{r(t_i)}} ,\breve{\varkappa }(t_i)\right) \cdot \curlyvee \left( s_{r(t_i)},\varkappa (t_i)\right) \right) \left\{ \left( \breve{\mu (t_i) ^{q}}\cdot \mu (t_i)^{q}\right) + \left( \breve{\nu (t_i)^{q}}\cdot \nu (t_i)^{q} \right) \right\} \right] =\textit{K}\left( \breve{{\mathcal {F}}},{\mathcal {F}}\right) . \end{aligned}$$

$$\square$$

#### Definition 10

Let $\texttt {Z}$ be a fixed set, $S=\left\{ s_{\theta }|\theta =1,2,...,\ell \right\}$ be a linguistic term set, for q-ROPFLSs ${\mathcal {F}}=\left\{ \left( \left( s_{r(t_i)},\varkappa (t_i)\right) ,\left\langle \mu (t_i), \nu (t_i)\right\rangle \right) \right\}$ and $\breve{{\mathcal {F}}}=\left\{ \left( \left( s_{\breve{r}(t_i)},\breve{\varkappa }(t_i)\right) ,\left\langle \breve{\mu }(t_i), \breve{\nu }(t_i)\right\rangle \right) \right\}$ on $\texttt {Z}$, the correlation coefficient of ${\mathcal {F}}$ and $\breve{{\mathcal {F}}}$ is presented by the following formula:

11 $$\begin{aligned} \begin{aligned}{}&\rho \left( {\mathcal {F}},\breve{{\mathcal {F}}}\right) =\frac{\textit{K}\left( {\mathcal {F}},\breve{{\mathcal {F}}}\right) }{\sqrt{\textit{E}\left( {\mathcal {F}}\right) }\sqrt{\textit{E}\left( \breve{{\mathcal {F}}}\right) }}\\&\quad = \frac{\sum \nolimits _{i=1}^{n}\left[ \left( \curlyvee \left( s_{r(t_i)}, \varkappa (t_i)\right) \cdot \curlyvee \left( s_{\breve{r(t_i)}},\breve{\varkappa }(t_i)\right) \right) \left\{ \left( \mu (t_i)^{q}\cdot \breve{\mu }(t_i)^{q}\right) +\left( \nu (t_i)^{q}\cdot \breve{\nu }(t_i)^{q}\right) \right\} \right] }{\sqrt{\sum \nolimits _{i=1}^{n}\left( \left( \curlyvee \left( s_{r(t_i)},\varkappa (t_i) \right) \right) ^2\mu (t_i)^{2q}+\left( \curlyvee \left( s_{r(t_i)},\varkappa (t_i)\right) \right) ^2\nu (t_i)^{2q}\right) } \sqrt{\sum \nolimits _{i=1}^{n}\left( \left( \curlyvee \left( s_{\breve{r}(t_i)}, \breve{\varkappa }(t_i)\right) \right) ^2\breve{\mu }(t_i)^{2q}+\left( \curlyvee \left( s_{\breve{r}(t_i)}, \breve{\varkappa } (t_i)\right) \right) ^2\breve{\nu }(t_i)^{2q}\right) }} \end{aligned} \end{aligned}$$

#### Theorem 2

The correlation coefficient between q-ROPFLSs ${\mathcal {F}}$ and $\breve{{\mathcal {F}}}$ holds the following properties:

1. $$\rho \left( {\mathcal {F}},\breve{{\mathcal {F}}}\right) =\rho \left( \breve{{\mathcal {F}}},{\mathcal {F}}\right)$$;
2. $$\rho \left( {\mathcal {F}},{\mathcal {F}}\right) =1$$;
3. $$0\le \rho \left( {\mathcal {F}},\breve{{\mathcal {F}}}\right) \le 1$$.

#### Proof

1. It is obvious, so we omit the proof.

2.

$$\begin{aligned}{} & {} \rho \left( {\mathcal {F}},{\mathcal {F}}\right) =\frac{\textit{K}\left( {\mathcal {F}},{\mathcal {F}}\right) }{\sqrt{\textit{E}\left( {\mathcal {F}}\right) }\sqrt{\textit{E}\left( {\mathcal {F}}\right) }} \\{} & {} \quad =\frac{\sum \limits _{i=1}^{n}\left[ \left( \curlyvee \left( s_{r(t_i)}, \varkappa (t_i)\right) \cdot \curlyvee \left( s_{r(t_i)},\varkappa (t_i)\right) \right) \left\{ \left( \mu (t_i)^{q}\cdot \mu (t_i)^{q}\right) +\left( \nu (t_i)^{q}\cdot \nu (t_i)^{q}\right) \right\} \right] }{\sqrt{\sum \limits _{i=1}^{n}\left( \left( \curlyvee \left( s_{r(t_i)},\varkappa (t_i)\right) \right) ^2\mu (t_i)^{2q}+\left( \curlyvee \left( s_{r(t_i)},\varkappa (t_i)\right) \right) ^2\nu (t_i)^{2q}\right) } \sqrt{\sum \limits _{i=1}^{n}\left( \left( \curlyvee \left( s_{r(t_i)},\varkappa (t_i)\right) \right) ^2\mu (t_i)^{2q}+\left( \curlyvee \left( s_{r(t_i)},\varkappa (t_i)\right) \right) ^2\nu (t_i)^{2q}\right) }}\\{} & {} \quad = \frac{\sum \limits _{i=1}^{n}\left( \left( \curlyvee \left( s_{r(t_i)},\varkappa (t_i) \right) \right) ^2\mu (t_i)^{2q}+\left( \curlyvee \left( s_{r(t_i)},\varkappa (t_i)\right) \right) ^2\nu (t_i)^{2q}\right) }{\left( \sqrt{\sum \limits _{i=1}^{n}\left( \left( \curlyvee \left( s_{r(t_i)},\varkappa (t_i)\right) \right) ^2\mu (t_i)^{2q}+\left( \curlyvee \left( s_{r(t_i)},\varkappa (t_i)\right) \right) ^2\nu (t_i)^{2q} \right) }\right) ^2} =1 \end{aligned}$$

3. The inequality $0\le \rho \left( {\mathcal {F}},\breve{{\mathcal {F}}}\right)$ is obvious. Below let us prove $\rho \left( {\mathcal {F}},\breve{{\mathcal {F}}}\right) \le 1.$

$$\begin{aligned}{} & {} \textit{K}\left( {\mathcal {F}},\breve{{\mathcal {F}}}\right) =\frac{1}{n} \sum \limits _{i=1}^{n}\left[ \frac{1}{\ell ^2}\left( \curlyvee \left( s_{r(t_i)}, \varkappa (t_i)\right) \curlyvee \left( s_{\breve{r(t_i)}},\breve{\varkappa }(t_i)\right) \right) \left\{ \left( \mu (t_i)^{q}\breve{\mu (t_i)^{q}}\right) +\left( \nu (t_i)^{q}\breve{\nu (t_i)^{q}}\right) \right\} \right] \\{} & {} \quad =\frac{1}{n}\sum \limits _{i=1}^{n}\left[ \frac{1}{\ell ^2}\left\{ \curlyvee \left( s_{r(t_i)},\varkappa (t_i)\right) \mu (t_i)^{q}\curlyvee \left( s_{\breve{r(t_i)}},\breve{\varkappa }(t_i)\right) \breve{\mu (t_i)^{q}} +\curlyvee \left( s_{r(t_i)},\varkappa (t_i)\right) \nu (t_i)^{q}\curlyvee \left( s_{\breve{r}(t_i)}, \breve{\varkappa }(t_i)\right) \breve{\nu (t_i)^{q}}\right\} \right] \\{} & {} \quad =\frac{1}{n}\left( \frac{1}{\ell ^2}\left\{ \curlyvee \left( s_{r(t_1)},\varkappa (t_1)\right) \mu (t_1)^{q}\curlyvee \left( s_{\breve{r(t_1)}},\breve{\varkappa }(t_1)\right) \breve{\mu (t_1)^{q}}+\cdots + \left( s_{r(t_n)},\varkappa (t_n)\right) \mu (t_n)^{q}\curlyvee \left( s_{\breve{r(t_n)}},\breve{\varkappa }(t_n) \right) \breve{\mu (t_n)^{q}}\right. \right. \\{} & {} \qquad +\left. \left. \curlyvee \left( s_{r(t_1)},\varkappa (t_1)\right) \nu (t_1)^{q}\curlyvee \left( s_{\breve{r}(t_1)}, \breve{\varkappa }(t_1)\right) \breve{\nu (t_1)^{q}}+\cdots + \curlyvee \left( s_{r(t_n)},\varkappa (t_n)\right) \nu (t_n)^{q}\curlyvee \left( s_{\breve{r}(t_n)}, \breve{\varkappa }(t_n)\right) \breve{\nu (t_n)^{q}}\right\} \right) \end{aligned}$$

According to the Cauchy-Schwarz inequality: $\left( a_1b_1+a_2b_2+\cdots +a_nb_n \right) ^2\le \left( a^2_1+a^2_2+\cdots +a^2_n \right) \left( b^2_1+b^2_2+\cdots +b^2_n \right)$ where $a_i, \; b_i \in R, \; i=1,2,...,n.$

$$\begin{aligned}{} & {} \textit{K}\left( {\mathcal {F}},\breve{{\mathcal {F}}}\right) ^2\\{} & {} \quad \le \left[ \frac{1}{n}\left( \left( \frac{\curlyvee \left( s_{r(t_1)},\varkappa (t_1)\right) }{\ell } \right) ^2\mu (t_1)^{2q}\right) +\cdots +\frac{1}{n}\left( \left( \frac{\curlyvee \left( s_{r(t_n)},\varkappa (t_n)\right) }{\ell } \right) ^2\mu (t_n)^{2q}\right) \right. \\{} & {} \qquad + \left. +\frac{1}{n}\left( \left( \frac{\curlyvee \left( s_{r(t_1)},\varkappa (t_1)\right) }{\ell } \right) ^2\nu (t_1)^{2q}\right) +\cdots +\frac{1}{n}\left( \left( \frac{\curlyvee \left( s_{r(t_n)},\varkappa (t_n)\right) }{\ell } \right) ^2\nu (t_n)^{2q}\right) \right] \\{} & {} \qquad \times \left[ \frac{1}{n}\left( \left( \frac{\curlyvee \left( s_{\breve{r}(t_1)},\breve{\varkappa }(t_1)\right) }{\ell } \right) ^2\breve{\mu }(t_1)^{2q}\right) +\cdots +\frac{1}{n}\left( \left( \frac{\curlyvee \left( \breve{s}_{r(t_n)},\breve{\varkappa }(t_n)\right) }{\ell } \right) ^2\breve{\mu }(t_n)^{2q}\right) \right. \\{} & {} \qquad \left. +\frac{1}{n}\left( \left( \frac{\curlyvee \left( s_{\breve{r}(t_1)},\breve{\varkappa }(t_1)\right) }{\ell } \right) ^2\breve{\nu }(t_1)^{2q}\right) +\cdots +\frac{1}{n} \left( \left( \frac{\curlyvee \left( s_{\breve{r}(t_n)},\breve{\varkappa }(t_n)\right) }{\ell } \right) ^2\breve{\nu }(t_n)^{2q}\right) \right] \\{} & {} \quad =\frac{1}{n}\sum _{i=1}^{n}\left( \left( \frac{\curlyvee \left( s_{r(t_i)}, \varkappa (t_i)\right) }{\ell } \right) ^2\mu (t_i)^{2q}+\left( \frac{\curlyvee \left( s_{r(t_i)}, \varkappa (t_i)\right) }{\ell } \right) ^2\nu (t_i)^{2q}\right) \\{} & {} \qquad \times \frac{1}{n}\sum _{i=1}^{n}\left( \left( \frac{\curlyvee \left( s_{\breve{r}(t_i)}, \breve{\varkappa }(t_i)\right) }{\ell } \right) ^2\breve{\mu }(t_i)^{2q}+\left( \frac{\curlyvee \left( s_{\breve{r}(t_i)},\breve{\varkappa }(t_i)\right) }{\ell } \right) ^2\breve{\nu }(t_i)^{2q}\right) \\{} & {} \quad =\textit{E}\left( {\mathcal {F}}\right) \times \textit{E}\left( \breve{{\mathcal {F}}}\right) . \end{aligned}$$

Therefore, $\textit{K}\left( {\mathcal {F}},\breve{{\mathcal {F}}}\right) \le \sqrt{\textit{E}\left( {\mathcal {F}}\right) } \sqrt{\textit{E}\left( \breve{{\mathcal {F}}}\right) },$ $0\le \rho \left( {\mathcal {F}},\breve{{\mathcal {F}}}\right) \le 1$ which means the correlation coefficient of two q-ROPFLSs lies between $\left[ 0,1 \right]$. $\square$

#### Example 1

Let ${\mathcal {F}}_1= \left\{ \left( \left( s_{4},0.0000\right) ,\left\langle 0.5853 ,0.3037 \right\rangle \right) , \left( \left( s_{5},0.0000\right) ,\left\langle 0.6810,0.1913 \right\rangle \right) ,\right. \left. \left( \left( s_{1},0.3333\right) ,\left\langle 0.6388,0.4380 \right\rangle \right) ,\left( \left( s_{2},-0.3333\right) ,\left\langle 0.3405,0.2520 \right\rangle \right) \right\}$ and ${\mathcal {F}}_2=\left\{ \left( \left( s_{3},0.0000\right) ,\left\langle 0.4059 ,0.2714 \right\rangle \right) , \left( \left( s_{6},0.3333\right) , \left\langle 0.2431, 0.5313\right\rangle \right) ,\left( \left( s_{5},0.0000\right) ,\left\langle 0.4059, 0.4000\right\rangle \right) ,\left( \left( s_{6},0.3333\right) ,\left\langle 0.6718 , 0.6316\right\rangle \right) \right\}$ be two q-ROPFTLSs in $\texttt {Z}=\left\{ t_1,t_2,t_3,t_4 \right\} .$ Then, from Eq. (11), we get $\rho \left( {\mathcal {F}}_1,{\mathcal {F}}_2\right) =0.2103.$

### Weighted covariance and correlation coefficient of q-ROPFLSs

The weighted correlation measure links the use of subject-assigned weights to the calculation of a correlation measure between two variables. The weights might either be readily available or selected by the researcher to meet a specific demand. For instance, if the number of estimates for each topic varies, it is customary to use these numbers as weights and calculate the correlation between the two variables. It has been shown that sporadic disagreements about distinct items may be associated with different weights. Consequently, while calculating the correlation coefficient between q-ROPFLSs, we shall consider the weighted impact. Within the framework of q-ROPFLS, we build a weighted correlation coefficient in the present part.

Suppose that the weight associated with each element $t_i$ is $\varpi _i$, where $\varpi _i \in [0,1](i=1,2,...,n)$ and $\sum \limits _{i=1}^{n}\varpi _i =1.$ Then

1. The weighted information energy of ${\mathcal {F}}$ is given by:
  12 $$\begin{aligned} {\textit{E}}_{\varpi }\left( {\mathcal {F}}\right) =\sum _{i=1}^{n}\varpi _i\left( \left( \frac{\curlyvee \left( s_{r(t_i)},\varkappa (t_i)\right) }{\ell } \right) ^2\mu (t_i)^{2q}+\left( \frac{\curlyvee \left( s_{r(t_i)},\varkappa (t_i)\right) }{\ell } \right) ^2\nu (t_i)^{2q}\right) . \end{aligned}$$
2. The weighted covariance of ${\mathcal {F}}$ and $\breve{{\mathcal {F}}}$ is presented by:
  13 $$\begin{aligned} {\textit{K}}_{\varpi }\left( {\mathcal {F}},\breve{{\mathcal {F}}}\right) =\sum _{i=1}^{n}{\varpi }_{i}\left[ \frac{1}{\ell ^2}\left( \curlyvee \left( s_{r(t_i)}, \varkappa (t_i)\right) \cdot \curlyvee \left( s_{\breve{r(t_i)}},\breve{\varkappa }(t_i)\right) \right) \left\{ \left( \mu (t_i)^{q}\cdot \breve{\mu (t_i)^{q}}\right) +\left( \nu (t_i)^{q}\cdot \breve{\nu (t_i)^{q}}\right) \right\} \right] . \end{aligned}$$
3. The weighted correlation coefficient of ${\mathcal {F}}$ and $\breve{{\mathcal {F}}}$ is given by:
  14 $$\begin{aligned} \begin{aligned}{}&\rho _{\varpi }\left( {\mathcal {F}},\breve{{\mathcal {F}}}\right) =\frac{{\textit{K}}_{\varpi } \left( {\mathcal {F}},\breve{{\mathcal {F}}}\right) }{\sqrt{{\textit{E}}_{\varpi }\left( {\mathcal {F}}\right) }\sqrt{{\textit{E}}_{\varpi }\left( \breve{{\mathcal {F}}}\right) }}\\&\quad = \frac{\sum \nolimits _{i=1}^{n}{\varpi }_{i}\left[ \left( \curlyvee \left( s_{r(t_i)}, \varkappa (t_i)\right) \cdot \curlyvee \left( s_{\breve{r(t_i)}},\breve{\varkappa }(t_i)\right) \right) \left\{ \left( \mu (t_i)^{q}\cdot \breve{\mu }(t_i)^{q}\right) +\left( \nu (t_i)^{q}\cdot \breve{\nu }(t_i)^{q}\right) \right\} \right] }{\sqrt{\sum \nolimits _{i=1}^{n}{\varpi }_{i}\left( \left( \curlyvee \left( s_{r(t_i)}, \varkappa (t_i)\right) \right) ^2\mu (t_i)^{2q}+\left( \curlyvee \left( s_{r(t_i)},\varkappa (t_i) \right) \right) ^2\nu (t_i)^{2q}\right) } \sqrt{\sum \nolimits _{i=1}^{n}{\varpi }_{i}\left( \left( \curlyvee \left( s_{\breve{r}(t_i)}, \breve{\varkappa }(t_i)\right) \right) ^2\breve{\mu }(t_i)^{2q}+\left( \curlyvee \left( s_{\breve{r}(t_i)}, \breve{\varkappa } (t_i)\right) \right) ^2\breve{\nu }(t_i)^{2q}\right) }} \end{aligned} \end{aligned}$$

#### Remark 3

For ${\varpi }=\left( \frac{1}{n},\frac{1}{n},\cdots \frac{1}{n} \right) ^T$, the weighted correlation coefficient reduces to correlation coefficient i.e., $\rho _{\varpi }\left( {\mathcal {F}},\breve{{\mathcal {F}}}\right) =\rho \left( {\mathcal {F}},\breve{{\mathcal {F}}}\right)$.

#### Theorem 4

The weighted correlation coefficient between q-ROPFLSs ${\mathcal {F}}$ and $\breve{{\mathcal {F}}}$ contains the following properties:

1. $$\rho _{\varpi }\left( {\mathcal {F}},\breve{{\mathcal {F}}}\right) =\rho _{\varpi } \left( \breve{{\mathcal {F}}},{\mathcal {F}}\right)$$;
2. $$\rho _{\varpi }\left( {\mathcal {F}},{\mathcal {F}}\right) =1$$;
3. $$0\le \rho _{\varpi }\left( {\mathcal {F}},\breve{{\mathcal {F}}}\right) \le 1$$.

#### Proof

1. It is obvious, so we omit the proof.

2.

$$\begin{aligned}{} & {} \rho _{\varpi }\left( {\mathcal {F}},{\mathcal {F}}\right) =\frac{{\textit{K}}_{\varpi } \left( {\mathcal {F}},{\mathcal {F}}\right) }{\sqrt{{\textit{E}}_{\varpi }\left( {\mathcal {F}}\right) }\sqrt{{\textit{E}}_{\varpi }\left( {\mathcal {F}}\right) }} \\{} & {} \quad =\frac{\sum \nolimits _{i=1}^{n}{\varpi }_{i}\left[ \left( \curlyvee \left( s_{r(t_i)}, \varkappa (t_i)\right) \cdot \curlyvee \left( s_{r(t_i)},\varkappa (t_i)\right) \right) \left\{ \left( \mu (t_i)^{q}\cdot \mu (t_i)^{q}\right) +\left( \nu (t_i)^{q}\cdot \nu (t_i)^{q}\right) \right\} \right] }{\sqrt{\sum \nolimits _{i=1}^{n}{\varpi }_{i}\left( \left( \curlyvee \left( s_{r(t_i)}, \varkappa (t_i)\right) \right) ^2\mu (t_i)^{2q}+\left( \curlyvee \left( s_{r(t_i)},\varkappa (t_i)\right) \right) ^2\nu (t_i)^{2q}\right) } \sqrt{\sum \nolimits _{i=1}^{n}{\varpi }_{i}\left( \left( \curlyvee \left( s_{r(t_i)},\varkappa (t_i)\right) \right) ^2\mu (t_i)^{2q}+\left( \curlyvee \left( s_{r(t_i)},\varkappa (t_i)\right) \right) ^2\nu (t_i)^{2q}\right) }}\\{} & {} \quad = \frac{\sum \nolimits _{i=1}^{n}{\varpi }_{i}\left( \left( \curlyvee \left( s_{r(t_i)}, \varkappa (t_i)\right) \right) ^2\mu (t_i)^{2q}+\left( \curlyvee \left( s_{r(t_i)},\varkappa (t_i) \right) \right) ^2\nu (t_i)^{2q}\right) }{\left( \sqrt{\sum \nolimits _{i=1}^{n}{\varpi }_{i}\left( \left( \curlyvee \left( s_{r(t_i)}, \varkappa (t_i)\right) \right) ^2\mu (t_i)^{2q}+\left( \curlyvee \left( s_{r(t_i)},\varkappa (t_i)\right) \right) ^2\nu (t_i)^{2q}\right) }\right) ^2} =1 \end{aligned}$$

3. The inequality $0\le \rho _{\varpi }\left( {\mathcal {F}},\breve{{\mathcal {F}}}\right)$ is obvious. Therefore, we need only to prove $\rho _{\varpi }\left( {\mathcal {F}},\breve{{\mathcal {F}}}\right) \le 1.$

$$\begin{aligned}{} & {} {\textit{K}}_{\varpi }\left( {\mathcal {F}},\breve{{\mathcal {F}}}\right) =\sum \limits _{i=1}^{n} {\varpi }_{i}\left[ \frac{1}{\ell ^2}\left( \curlyvee \left( s_{r(t_i)}, \varkappa (t_i)\right) \curlyvee \left( s_{\breve{r(t_i)}},\breve{\varkappa }(t_i)\right) \right) \left\{ \left( \mu (t_i)^{q}\breve{\mu (t_i)^{q}}\right) +\left( \nu (t_i)^{q}\breve{\nu (t_i)^{q}}\right) \right\} \right] \\{} & {} \quad = \sum \limits _{i=1}^{n}{\varpi }_{i}\left[ \frac{1}{\ell ^2}\left\{ \curlyvee \left( s_{r(t_i)},\varkappa (t_i)\right) \mu (t_i)^{q}\curlyvee \left( s_{\breve{r(t_i)}},\breve{\varkappa }(t_i)\right) \breve{\mu (t_i)^{q}} +\curlyvee \left( s_{r(t_i)},\varkappa (t_i)\right) \nu (t_i)^{q}\curlyvee \left( s_{\breve{r}(t_i)}, \breve{\varkappa }(t_i)\right) \breve{\nu (t_i)^{q}}\right\} \right] \\{} & {} \quad =\left( \frac{1}{\ell ^2}\left\{ {\varpi }_{1}\curlyvee \left( s_{r(t_1)},\varkappa (t_1)\right) \mu (t_1)^{q}\curlyvee \left( s_{\breve{r(t_1)}},\breve{\varkappa }(t_1)\right) \breve{\mu (t_1)^{q}}+\cdots + {\varpi }_{n}\curlyvee \left( s_{r(t_n)},\varkappa (t_n)\right) \mu (t_n)^{q}\curlyvee \left( s_{\breve{r(t_n)}},\breve{\varkappa }(t_n)\right) \breve{\mu (t_n)^{q}}\right. \right. \\{} & {} \qquad + \left. \left. {\varpi }_{1}\curlyvee \left( s_{r(t_1)},\varkappa (t_1)\right) \nu (t_1)^{q}\curlyvee \left( s_{\breve{r}(t_1)}, \breve{\varkappa }(t_1)\right) \breve{\nu (t_1)^{q}}+\cdots + {\varpi }_{n}\curlyvee \left( s_{r(t_n)},\varkappa (t_n)\right) \nu (t_n)^{q}\curlyvee \left( s_{\breve{r}(t_n)}, \breve{\varkappa }(t_n)\right) \breve{\nu (t_n)^{q}}\right\} \right) \end{aligned}$$

According to the Cauchy-Schwarz inequality: $\left( a_1b_1+a_2b_2+\cdots +a_nb_n \right) ^2\le \left( a^2_1+a^2_2+\cdots +a^2_n \right) \left( b^2_1+b^2_2+\cdots +b^2_n \right)$ where $a_i, \; b_i \in R, \; i=1,2,...,n.$

$$\begin{aligned}{} & {} {\textit{K}}_{\varpi }\left( {\mathcal {F}},\breve{{\mathcal {F}}}\right) ^2\\{} & {} \quad \le \left[ {\varpi }_{1}\left( \left( \frac{\curlyvee \left( s_{r(t_1)},\varkappa (t_1)\right) }{\ell } \right) ^2\mu (t_1)^{2q}\right) +\cdots +{\varpi }_{n}\left( \left( \frac{\curlyvee \left( s_{r(t_n)},\varkappa (t_n)\right) }{\ell } \right) ^2\mu (t_n)^{2q}\right) \right. \\{} & {} \qquad + \left. +{\varpi }_{1}\left( \left( \frac{\curlyvee \left( s_{r(t_1)},\varkappa (t_1)\right) }{\ell } \right) ^2\nu (t_1)^{2q}\right) +\cdots +{\varpi }_{n}\left( \left( \frac{\curlyvee \left( s_{r(t_n)}, \varkappa (t_n)\right) }{\ell } \right) ^2\nu (t_n)^{2q}\right) \right] \\{} & {} \qquad \times \left[ {\varpi }_{1}\left( \left( \frac{\curlyvee \left( s_{\breve{r}(t_1)},\breve{\varkappa }(t_1)\right) }{\ell } \right) ^2\breve{\mu }(t_1)^{2q}\right) +\cdots +{\varpi }_{n}\left( \left( \frac{\curlyvee \left( \breve{s}_{r(t_n)},\breve{\varkappa }(t_n)\right) }{\ell } \right) ^2\breve{\mu }(t_n)^{2q}\right) +\right. \\{} & {} \quad \left. +{\varpi }_{1}\left( \left( \frac{\curlyvee \left( s_{\breve{r}(t_1)},\breve{\varkappa } (t_1)\right) }{\ell } \right) ^2\breve{\nu }(t_1)^{2q}\right) +\cdots +{\varpi }_{n} \left( \left( \frac{\curlyvee \left( s_{\breve{r}(t_n)},\breve{\varkappa }(t_n)\right) }{\ell } \right) ^2\breve{\nu }(t_n)^{2q}\right) \right] \\{} & {} \quad = \sum \limits _{i=1}^{n}{\varpi }_{i}\left( \left( \frac{\curlyvee \left( s_{r(t_i)}, \varkappa (t_i)\right) }{\ell } \right) ^2\mu (t_i)^{2q}+\left( \frac{\curlyvee \left( s_{r(t_i)}, \varkappa (t_i)\right) }{\ell } \right) ^2\nu (t_i)^{2q}\right) \\{} & {} \qquad \times \sum \limits _{i=1}^{n}{\varpi }_{i}\left( \left( \frac{\curlyvee \left( s_{\breve{r}(t_i)}, \breve{\varkappa }(t_i)\right) }{\ell } \right) ^2\breve{\mu }(t_i)^{2q}+\left( \frac{\curlyvee \left( s_{\breve{r} (t_i)},\breve{\varkappa }(t_i)\right) }{\ell } \right) ^2\breve{\nu }(t_i)^{2q}\right) \\{} & {} \quad ={\textit{E}}_{\varpi }\left( {\mathcal {F}}\right) \times {\textit{E}}_ {\varpi }\left( \breve{{\mathcal {F}}}\right) . \end{aligned}$$

Therefore, ${\textit{K}}_{\varpi }\left( {\mathcal {F}},\breve{{\mathcal {F}}}\right) \le \sqrt{{\textit{E}}_{\varpi }\left( {\mathcal {F}}\right) } \sqrt{{\textit{E}}_{\varpi }\left( \breve{{\mathcal {F}}}\right) },$ $0\le {\rho }_{\varpi }\left( {\mathcal {F}},\breve{{\mathcal {F}}}\right) \le 1$ which means the correlation coefficient of two q-ROPFLSs lies between $\left[ 0,1 \right]$. $\square$

#### Example 2

Let ${\mathcal {F}}_1= \left\{ \left( \left( s_{4},0.0000\right) ,\left\langle 0.5853 ,0.3037 \right\rangle \right) , \left( \left( s_{5},0.0000\right) ,\left\langle 0.6810,0.1913 \right\rangle \right) ,\right. \left. \left( \left( s_{1},0.3333\right) ,\left\langle 0.6388,0.4380 \right\rangle \right) ,\left( \left( s_{2},-0.3333\right) ,\left\langle 0.3405,0.2520 \right\rangle \right) \right\}$ and ${\mathcal {F}}_2=\left\{ \left( \left( s_{3},0.0000\right) ,\left\langle 0.4059 ,0.2714 \right\rangle \right) ,\right. \left. \left( \left( s_{6},0.3333\right) , \left\langle 0.2431, 0.5313\right\rangle \right) ,\left( \left( s_{5},0.0000\right) ,\left\langle 0.4059, 0.4000\right\rangle \right) ,\left( \left( s_{6},0.3333\right) ,\left\langle 0.6718 , 0.6316\right\rangle \right) \right\}$ be two QROPFTLSs in $\texttt {Z}=\left\{ t_1,t_2,t_3,t_4 \right\} ,$ $w=\left\{ 0.2353, 0.2727, 0.1912, 0.3008\right\}$ be the weight vector of $t_i(i=1,2,3,4)$. Then, from Eq. (14), we get $\rho _{\varpi }\left( {\mathcal {F}}_1,{\mathcal {F}}_2\right) =0.1224.$

## Clustering algorithm under q-ROPFL environment

Based on the q-rung orthopair fuzzy clustering method^36^ and the previously designed correlation coefficient formulas for q-ROPFLSs, we construct an approach for clustering in q-ROPFL environment. Firstly, the following ideas are introduced:

### Theoretical development

#### Definition 11

Let ${{\mathcal {F}}}_j\left( j=1,2,...,m \right)$ be *m* q-ROPFLSs, then $C=\left( \rho _{ij} \right) _{m \times m}$ is called a correlation matrix, where $\rho _{ij}=\rho \left( {\mathcal {F}}_i,{\mathcal {F}}_j\right)$ denotes the correlation coefficient of ${{\mathcal {F}}}_i$ and ${{\mathcal {F}}}_j$, which meets the following characteristics:

1. $$0\le \rho _{ij}\le 1 \; \forall \; i,j=1,2,...,m;$$
2. $\rho _{ij}=1$ if and only if ${{\mathcal {F}}}_i={{\mathcal {F}}}_j$;
3. $$\rho _{ij}=\rho _{ji}\; \forall \; i,j=1,2,...,m.$$

#### Definition 12

Let $C=\left( \rho _{ij} \right) _{m \times m}$ be a correlation matrix, if $C^2=C\circ C=\left( \ddot{\rho _{ij}}\right) _{m \times m}$ is called a composition matrix of *C*, where

15 $$\begin{aligned} \ddot{\rho _{ij}}=\max \limits _{k}\left\{ \min \left\{ \rho _{ik},\rho _{kj}\right\} \right\} ,\; i,j=1,2,...,m. \end{aligned}$$

Based on Definition 12, we have

#### Theorem 5

Let $C=\left( \rho _{ij} \right) _{m \times m}$ be a correlation matrix, then the composition matrix $C^2$ is also a correlation matrix.

#### Proof

1. Since *C* is a correlation matrix, then $0\le \rho _{ij}\le 1 \; \forall \; i,j=1,2,...,m.$ Thus $0\le \ddot{\rho _{ij}}=\max \limits _{k}\left\{ \min \left\{ \rho _{ik},\rho _{kj}\right\} \right\} \le 1\; \forall \; i,j=1,2,...,m.$
2. Since $\rho _{ij}=1$ if and only if ${{\mathcal {F}}}_i={{\mathcal {F}}}_j$, then $\ddot{\rho _{ij}}=\max \limits _{k}\left\{ \min \left\{ \rho _{ik},\rho _{kj}\right\} \right\} =1$ if and only if ${{\mathcal {F}}}_i={{\mathcal {F}}}_k={{\mathcal {F}}}_j$ for some *k*.
3. Since $$\rho _{ij}=\rho _{ji}\; \forall \; i,j=1,2,...,m,$$ then $$\ddot{\rho _{ij}}=\max \limits _{k}\left\{ \min \left\{ \rho _{ik},\rho _{kj}\right\} \right\} = \ddot{\rho _{ij}}=\max \limits _{k}\left\{ \min \left\{ \rho _{ki},\rho _{jk}\right\} \right\} = \ddot{\rho _{ij}}=\max \limits _{k}\left\{ \min \left\{ \rho _{jk},\rho _{ki}\right\} \right\} =\ddot{\rho _{ji}}\; \forall \; i,j=1,2,...,m.$$

Thus, we complete the proof of Theorem 5. $\square$

#### Theorem 6

Let $C=\left( \rho _{ij} \right) _{m \times m}$ be a correlation matrix, then for any non-negative integer *k*, the composition matrix ${C^{2}}^{k+1}$ acquired from

16 $$\begin{aligned} {C^{2}}^{k+1}={C^{2}}^k\circ {C^{2}}^k \end{aligned}$$

is also a correlation matrix.

#### Proof

We prove this by using mathematical induction method.

If $k=0$, then $C^2=C \circ C,$ thus, by Theorem 5, $C^2$ is a correlation matrix.

Suppose for $k=\acute{k}$ Eq. (16) holds, i.e., ${C^{2}}^{\acute{k}+1}={C^{2}}^{\acute{k}} \circ {C^{2}}^{\acute{k}}$ and ${C^{2}}^{\acute{k}+1}$ is a correlation matrix.

For $k=\acute{k}+1$ , we have

$${C^{2}}^{k+1}={C^{2}}^{(\acute{k}+1)+1}=\left( {C^{2}}^{\acute{k}+1}\right) ^2={C^{2}}^{\acute{k}+1}\circ {C^{2}}^{\acute{k}+1}$$.

Thus, by Theorem 5, ${C^{2}}^{(\acute{k}+1)+1}$ is also a correlation matrix. $\square$

#### Definition 13

Let $C=\left( \rho _{ij} \right) _{m \times m}$ be a correlation matrix, if $C^2\subseteq C,$ i.e.,

17 $$\begin{aligned} \max \limits _{k}\left\{ \min \left\{ \rho _{ik},\rho _{kj}\right\} \right\} \le \rho _{ij}\; i,j=1,2,...,m, \end{aligned}$$

then *C* is called an equivalent correlation matrix.

#### Lemma 1

^37^ Let $C=\left( \rho _{ij} \right) _{m \times m}$ be a correlation matrix, then after the finite times of composition:

18 $$\begin{aligned} C \rightarrow C^2 \rightarrow C^4 \rightarrow ... \rightarrow {C^2}^k \rightarrow ... \end{aligned}$$

There exists a positive integer *k* such that ${C^2}^k={C^2}^{k+1},$ and ${C^2}^k$ is also an equivalent correlation matrix.

#### Theorem 7

An equivalent correlation matrix can be derived after the finite times of composition from the correlation matrix $C=\left( \rho _{ij} \right) _{m \times m}.$

#### Proof

If $C=\left( \rho _{ij} \right) _{m \times m}$ is equivalent correlation matrix. Then the theorem is true obviously. If not, then by Lemma 1, there must exist some positive integer *k* such that ${C^2}^k={C^2}^{k+1},$ and ${C^2}^k$ is also an equivalent correlation matrix. Since, ${C^2}^k={C^2}^{k+1},$ then $\left( {C^2}^k\right) ^2 \subseteq {C^2}^{k+1},$ i.e., ${C^2}^k$ is an equivalent correlation matrix. $\square$

#### Definition 14

Let $C=\left( \rho _{ij} \right) _{m \times m}$ be a correlation matrix, then $C_{\zeta }=\left( \zeta \rho _{ij} \right) _{m \times m}$ is called $\zeta -$cutting matrix of *C*, where

19 $$\begin{aligned} \zeta \rho _{ij}=\left. {\left\{ \begin{array}{ll} 0, &{} \text{ if } \rho _{ij} < \zeta \\ 1, &{} \text{ if } \rho _{ij} \ge \zeta , \end{array}\right. }\right. i,j=1,2,...,m \end{aligned}$$

and $\zeta$ is the confidence level with $\zeta \in [0,1]$.

### Clustering algorithm

This segment is devoted to put forward the MCGDM model on the basis of propounding theory.

Let ${\mathcal {O}}=\left\{ {\mathcal {O}}_1,{\mathcal {O}}_2,...,{\mathcal {O}}_m \right\}$ be the alternatives set, ${\mathcal {C}}=\left\{ {\mathcal {C}}_1,{\mathcal {C}}_2,...,{\mathcal {C}}_n \right\}$ be the criteria set for each alternative and $\varpi =\left\{ \varpi _1,\varpi _2,...,\varpi _n \right\}$ be the weight vector of criteria set ${\mathcal {C}}$. The weight vector $\varpi$ is utilized to depict the importance of different criteria in the process of decision making, where $\sum \limits _{j=1}^{n}\varpi _j=1$ and $\varpi _j \in \left[ 0,1 \right]$. The invited ‘*p*’ DMs ${\mathcal {D}}=\left\{ {\mathcal {D}}_1,{\mathcal {D}}_2,...,{\mathcal {D}}_p \right\}$ evaluate each alternative ${\mathcal {O}}_i$ under the criteria ${\mathcal {C}}_j$ in terms of q-ROPFLNs $\partial ^k_{ij}$. The main steps involved in the proposed model are manifested as below:

Step 1: Collect the q-ROPFL experimental data set provided by each ${\mathcal {D}}_k$ $(k=1,2,...,p)$ using the LTS *S*, which includes information about the alternatives described by their relevant characteristics/ criteria. The assessment information of ${\mathcal {D}}_k$ can be described in the form of a decision matrix as
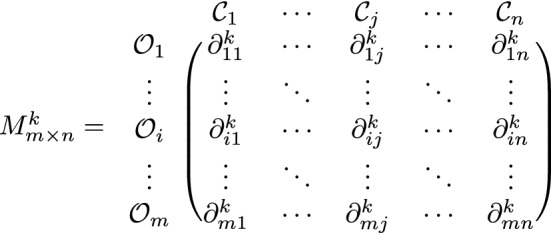


where $\partial ^k_{ij}$ is the *ij*th q-ROPFLN provided by *k*th DM to which alternative ${\mathcal {O}}_i$ satisfies the criteria ${\mathcal {C}}_j$.

Step 2: Apply the q-ROPFLA operator Eq. (6) to aggregate the individual matrices into collective decision matrix $M=\left[ \partial _{ij} \right] _{m \times n}$,
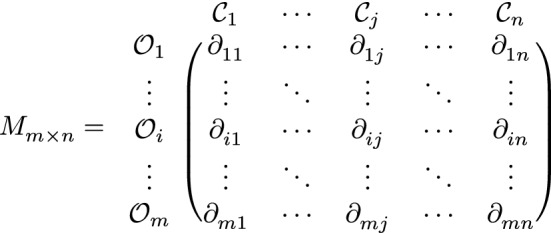


where $\partial _{ij}$ can be determined as follows:

20 $$\begin{aligned} \partial _{ij} =\left( \curlywedge \left( \frac{1}{p}\sum _{k=1}^{p}\curlyvee \left( s_{r^k_{ij}},{\varkappa }^k_{ij}\right) \right) , \left\langle \left( 1-\prod _{k=1}^{p}\left( 1- \left( {\mu }^k_{ij}\right) ^q \right) ^{\frac{1}{p}}\right) ^{1/q}, \prod _{k=1}^{p}\left( \nu ^k_{ij}\right) ^{\frac{1}{p}}\right\rangle \right) . \end{aligned}$$

Step 3: Based on maximizing deviation model^19^, determine the weights of criteria $\varpi =\left( \varpi _1,\varpi _2,...,\varpi _n \right) .$

Step 4: According to Eq. (14), compute the weighted correlation coefficient between ${{\mathcal {F}}}_i$ and ${{\mathcal {F}}}_j$, and then build the correlation matrix $C=\left( \rho _{ij} \right) _{m \times m}$ where $\rho _{ij} =\rho \left( {{\mathcal {F}}}_i,{{\mathcal {F}}}_j\right)$ $\left( i,j=1,2,...,m\right) .$

Step 5: Check whether $C=\left( \rho _{ij} \right) _{m \times m}$ is an equivalent correlation matrix, i.e., $C^2\subseteq C$, where

$$\begin{aligned} C^2=C\circ C=\left( \ddot{\rho _{ij}}\right) _{m \times m},\;\ddot{\rho _{ij}}=\max \limits _{k}\left\{ \min \left\{ \rho _{ik},\rho _{kj}\right\} \right\} ,\; i,j=1,2,...,m. \end{aligned}$$

If it does not hold, we construct the equivalent correlation matrix ${C^2}^k:$

$$\begin{aligned} C \rightarrow C^2 \rightarrow C^4 \rightarrow ... \rightarrow {C^2}^k \rightarrow ..., \text { until } {C^2}^k={C^2}^{k+1}. \end{aligned}$$

Step 6: To categorise the q-ROPFLSs ${{\mathcal {F}}}_i\left( i=1,2,...,m \right)$, we create a $\zeta$-cutting matrix $C_{\zeta }=\left( \zeta \rho _{ij} \right) _{m \times m}$ according to Definition 14 with a confidence level $\zeta$. If all components of the *i*th line (column) in $C_{\zeta }$ match those of the *j*th line (column) in $C_{\zeta }$, then the q-ROPFLSs ${{\mathcal {F}}}_i$ and ${{\mathcal {F}}}_j$ are of the same type. This practice enables us to categorise these *m* q-ROPLSs ${{\mathcal {F}}}_i (i= 1,2,...,m)$.

**Figure 2:**
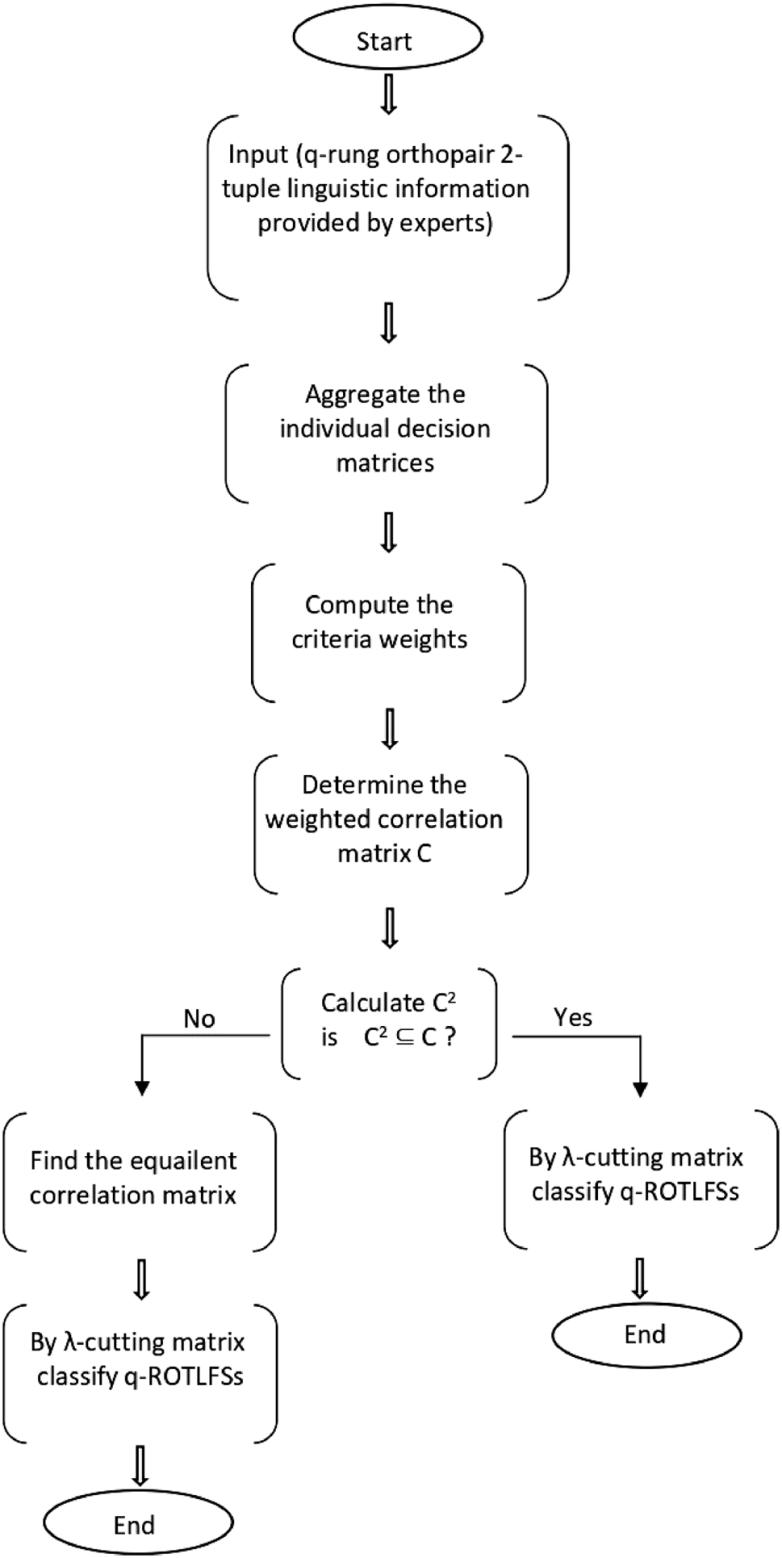
Flow chart of the developed clustering algorithm.

Figure 2 provides a flowchart depiction of the method for easier comprehension.

## Applications and analysis

This section provides some examples to explain the applicability and validity of the proposed clustering algorithm.

### Illustrated example

Software assessment and categorization is becoming increasingly essential issue in all areas of human activity. Industrial production, service delivery, and corporate administration are all strongly reliant on software, which is becoming more sophisticated and costly^38^. A CASE tool to help software development in a CIM context must be chosen among those available on the market. CIM software is often in charge of production planning, control, and monitoring^39^.

We do clustering for various kinds of CIM software ${\mathcal {O}}_i(i = 1,2,..., 7)$ on the market to better assess them based on four criteria: ${{\mathcal {C}}}_1$: functionality, ${{\mathcal {C}}}_2$: usability, ${{\mathcal {C}}}_3$: portability, and ${{\mathcal {C}}}_4$: maturity. Given that the specialists who do such an examination have varying backgrounds and degrees of knowledge, abilities, experience, personality, and so on, the evaluation information may differ. The experts are provided with the LTS

$$\begin{aligned} S=\left\{ \begin{array}{c} s_1 = \text {extremely poor}, s_2 = \text {very poor}, s_3 = \text {poor}, s_4 = \text {medium}, s_5 = \text {good}, s_6 = \text {very good}, \\ s_7 = \text {extremely good} \end{array}\right\} . \end{aligned}$$

The assessment information is represented by the q-ROPLSs and enlisted in Tables 1, 2 and 3 to clearly indicate the disparities in the judgments of experts.

**Table 1:** q-ROPFL data provided by ${\mathcal {D}}_1$.

	${\mathcal {C}}_1$	${\mathcal {C}}_2$	${\mathcal {C}}_3$	${\mathcal {C}}_4$
${\mathcal {O}}_1$	$\left( \left( s_{4},0\right) ,\left\langle 0.7, 0.2\right\rangle \right)$	$\left( \left( s_{5},0\right) ,\left\langle 0.8, 0.1\right\rangle \right)$	$\left( \left( s_{1},0\right) ,\left\langle 0.7, 0.3\right\rangle \right)$	$\left( \left( s_{3},0\right) ,\left\langle 0.4, 0.2\right\rangle \right)$
${\mathcal {O}}_2$	$\left( \left( s_{3},0\right) ,\left\langle 0.5, 0.2\right\rangle \right)$	$\left( \left( s_{6},0\right) ,\left\langle 0.2, 0.5\right\rangle \right)$	$\left( \left( s_{5},0\right) ,\left\langle 0.4, 0.4\right\rangle \right)$	$\left( \left( s_{7},0\right) ,\left\langle 0.6, 0.7\right\rangle \right)$
${\mathcal {O}}_3$	$\left( \left( s_{6},0\right) ,\left\langle 0.5, 0.4\right\rangle \right)$	$\left( \left( s_{2},0\right) ,\left\langle 0.3, 0.8\right\rangle \right)$	$\left( \left( s_{1},0\right) ,\left\langle 0.6, 0.5\right\rangle \right)$	$\left( \left( s_{2},0\right) ,\left\langle 0.3, 0.8\right\rangle \right)$
${\mathcal {O}}_4$	$\left( \left( s_{7},0\right) ,\left\langle 0.5, 0.5\right\rangle \right)$	$\left( \left( s_{1},0\right) ,\left\langle 0.6, 0.2\right\rangle \right)$	$\left( \left( s_{4},0\right) ,\left\langle 0.2, 0.7\right\rangle \right)$	$\left( \left( s_{3},0\right) ,\left\langle 0.4, 0.8\right\rangle \right)$
${\mathcal {O}}_5$	$\left( \left( s_{6},0\right) ,\left\langle 0.8, 0.4\right\rangle \right)$	$\left( \left( s_{4},0\right) ,\left\langle 0.3, 0.2\right\rangle \right)$	$\left( \left( s_{3},0\right) ,\left\langle 0.8, 0.3\right\rangle \right)$	$\left( \left( s_{5},0\right) ,\left\langle 0.6, 0.5\right\rangle \right)$
${\mathcal {O}}_6$	$\left( \left( s_{4},0\right) ,\left\langle 0.5, 0.3\right\rangle \right)$	$\left( \left( s_{5},0\right) ,\left\langle 0.6, 0.4\right\rangle \right)$	$\left( \left( s_{3},0\right) ,\left\langle 0.2, 0.7\right\rangle \right)$	$\left( \left( s_{1},0\right) ,\left\langle 0.3, 0.6\right\rangle \right)$
${\mathcal {O}}_7$	$\left( \left( s_{4},0\right) ,\left\langle 0.5, 0.5\right\rangle \right)$	$\left( \left( s_{6},0\right) ,\left\langle 0.7, 0.8\right\rangle \right)$	$\left( \left( s_{5},0\right) ,\left\langle 0.4, 0.6\right\rangle \right)$	$\left( \left( s_{2},0\right) ,\left\langle 0.5, 0.4\right\rangle \right)$

**Table 2:** q-ROPFL data provided by ${\mathcal {D}}_2$.

	${\mathcal {C}}_1$	${\mathcal {C}}_2$	${\mathcal {C}}_3$	${\mathcal {C}}_4$
${\mathcal {O}}_1$	$\left( \left( s_{3},0\right) ,\left\langle 0.6, 0.2\right\rangle \right)$	$\left( \left( s_{4},0\right) ,\left\langle 0.7, 0.1\right\rangle \right)$	$\left( \left( s_{1},0\right) ,\left\langle 0.7, 0.4\right\rangle \right)$	$\left( \left( s_{2},0\right) ,\left\langle 0.3, 0.4\right\rangle \right)$
${\mathcal {O}}_2$	$\left( \left( s_{2},0\right) ,\left\langle 0.4, 0.2\right\rangle \right)$	$\left( \left( s_{6},0\right) ,\left\langle 0.3, 0.5\right\rangle \right)$	$\left( \left( s_{4},0\right) ,\left\langle 0.2, 0.4\right\rangle \right)$	$\left( \left( s_{6},0\right) ,\left\langle 0.7, 0.6\right\rangle \right)$
${\mathcal {O}}_3$	$\left( \left( s_{5},0\right) ,\left\langle 0.4, 0.5\right\rangle \right)$	$\left( \left( s_{2},0\right) ,\left\langle 0.4, 0.8\right\rangle \right)$	$\left( \left( s_{2},0\right) ,\left\langle 0.5, 0.5\right\rangle \right)$	$\left( \left( s_{1},0\right) ,\left\langle 0.2, 0.8\right\rangle \right)$
${\mathcal {O}}_4$	$\left( \left( s_{6},0\right) ,\left\langle 0.4, 0.5\right\rangle \right)$	$\left( \left( s_{1},0\right) ,\left\langle 0.7, 0.2\right\rangle \right)$	$\left( \left( s_{3},0\right) ,\left\langle 0.3, 0.7\right\rangle \right)$	$\left( \left( s_{2},0\right) ,\left\langle 0.4, 0.7\right\rangle \right)$
${\mathcal {O}}_5$	$\left( \left( s_{5},0\right) ,\left\langle 0.7, 0.4\right\rangle \right)$	$\left( \left( s_{5},0\right) ,\left\langle 0.3, 0.3\right\rangle \right)$	$\left( \left( s_{2},0\right) ,\left\langle 0.7, 0.4\right\rangle \right)$	$\left( \left( s_{5},0\right) ,\left\langle 0.6, 0.5\right\rangle \right)$
${\mathcal {O}}_6$	$\left( \left( s_{3},0\right) ,\left\langle 0.4, 0.3\right\rangle \right)$	$\left( \left( s_{3},0\right) ,\left\langle 0.6, 0.5\right\rangle \right)$	$\left( \left( s_{2},0\right) ,\left\langle 0.3, 0.7\right\rangle \right)$	$\left( \left( s_{1},0\right) ,\left\langle 0.4, 0.6\right\rangle \right)$
${\mathcal {O}}_7$	$\left( \left( s_{3},0\right) ,\left\langle 0.5, 0.4\right\rangle \right)$	$\left( \left( s_{5},0\right) ,\left\langle 0.6, 0.8\right\rangle \right)$	$\left( \left( s_{4},0\right) ,\left\langle 0.5, 0.6\right\rangle \right)$	$\left( \left( s_{2},0\right) ,\left\langle 0.4, 0.4\right\rangle \right)$

**Table 3:** q-ROPFL data provided by ${\mathcal {D}}_3$.

	${\mathcal {C}}_1$	${\mathcal {C}}_2$	${\mathcal {C}}_3$	${\mathcal {C}}_4$
${\mathcal {O}}_1$	$\left( \left( s_{5},0\right) ,\left\langle 0.2, 0.7\right\rangle \right)$	$\left( \left( s_{6},0\right) ,\left\langle 0.1, 0.7\right\rangle \right)$	$\left( \left( s_{2},0\right) ,\left\langle 0.4, 0.7\right\rangle \right)$	$\left( \left( s_{3},0\right) ,\left\langle 0.3, 0.2\right\rangle \right)$
${\mathcal {O}}_2$	$\left( \left( s_{4},0\right) ,\left\langle 0.2, 0.5\right\rangle \right)$	$\left( \left( s_{7},0\right) ,\left\langle 0.2, 0.6\right\rangle \right)$	$\left( \left( s_{6},0\right) ,\left\langle 0.5, 0.4\right\rangle \right)$	$\left( \left( s_{6},0\right) ,\left\langle 0.7, 0.6\right\rangle \right)$
${\mathcal {O}}_3$	$\left( \left( s_{7},0\right) ,\left\langle 0.4, 0.4\right\rangle \right)$	$\left( \left( s_{3},0\right) ,\left\langle 0.8, 0.3\right\rangle \right)$	$\left( \left( s_{1},0\right) ,\left\langle 0.6, 0.5\right\rangle \right)$	$\left( \left( s_{3},0\right) ,\left\langle 0.7, 0.3\right\rangle \right)$
${\mathcal {O}}_4$	$\left( \left( s_{6},0\right) ,\left\langle 0.6, 0.5\right\rangle \right)$	$\left( \left( s_{1},0\right) ,\left\langle 0.6, 0.3\right\rangle \right)$	$\left( \left( s_{4},0\right) ,\left\langle 0.7, 0.4\right\rangle \right)$	$\left( \left( s_{3},0\right) ,\left\langle 0.5, 0.6\right\rangle \right)$
${\mathcal {O}}_5$	$\left( \left( s_{5},0\right) ,\left\langle 0.8, 0.4\right\rangle \right)$	$\left( \left( s_{4},0\right) ,\left\langle 0.4, 0.2\right\rangle \right)$	$\left( \left( s_{3},0\right) ,\left\langle 0.8, 0.3\right\rangle \right)$	$\left( \left( s_{6},0\right) ,\left\langle 0.6, 0.5\right\rangle \right)$
${\mathcal {O}}_6$	$\left( \left( s_{3},0\right) ,\left\langle 0.5, 0.2\right\rangle \right)$	$\left( \left( s_{3},0\right) ,\left\langle 0.6, 0.4\right\rangle \right)$	$\left( \left( s_{4},0\right) ,\left\langle 0.7, 0.6\right\rangle \right)$	$\left( \left( s_{1},0\right) ,\left\langle 0.6, 0.3\right\rangle \right)$
${\mathcal {O}}_7$	$\left( \left( s_{5},0\right) ,\left\langle 0.6, 0.5\right\rangle \right)$	$\left( \left( s_{5},0\right) ,\left\langle 0.8, 0.7\right\rangle \right)$	$\left( \left( s_{6},0\right) ,\left\langle 0.6, 0.6\right\rangle \right)$	$\left( \left( s_{3},0\right) ,\left\langle 0.5, 0.5\right\rangle \right)$

Now we proceed to follow the steps of the established algorithm as:

Step 1: The q-ROPFL decision matrices $M^{k}_{7 \times 4} (k = 1,2,3)$ are shown in Tables 1-3.

Step 2: Employ the q-ROPFLA operator Eq. (20) (taking $q=3$) to aggregate all the matrices into collective decision matrix (see Table 4).

**Table 4:** Aggregated q-ROPFL decision matrix.

	${\mathcal {C}}_1$	${\mathcal {C}}_2$	${\mathcal {C}}_3$	${\mathcal {C}}_4$
${\mathcal {O}}_1$	$\left( \left( s_{4},0.0000\right) ,\left\langle 0.5853 ,0.3037 \right\rangle \right)$	$\left( \left( s_{5},0.0000\right) ,\left\langle 0.6810,0.1913 \right\rangle \right)$	$\left( \left( s_{1},0.3333\right) ,\left\langle 0.6388,0.4380 \right\rangle \right)$	$\left( \left( s_{2},-0.3333\right) ,\left\langle 0.3405,0.2520 \right\rangle \right)$
${\mathcal {O}}_2$	$\left( \left( s_{3},0.0000\right) ,\left\langle 0.4059 ,0.2714 \right\rangle \right)$	$\left( \left( s_{6},0.3333\right) ,\left\langle 0.2431, 0.5313\right\rangle \right)$	$\left( \left( s_{5},0.0000\right) ,\left\langle 0.4059, 0.4000\right\rangle \right)$	$\left( \left( s_{6},0.3333\right) ,\left\langle 0.6718 , 0.6316\right\rangle \right)$
${\mathcal {O}}_3$	$\left( \left( s_{6},0.0000\right) ,\left\langle 0.4393, 0.4309\right\rangle \right)$	$\left( \left( s_{2},0.3333\right) ,\left\langle 0.6187 , 0.5769 \right\rangle \right)$	$\left( \left( s_{1},0.3333\right) ,\left\langle 0.5716, 0.5000\right\rangle \right)$	$\left( \left( s_{2},0.0000\right) ,\left\langle 0.5203,0.5769 \right\rangle \right)$
${\mathcal {O}}_4$	$\left( \left( s_{6},0.3333\right) ,\left\langle 0.5159 ,0.5000 \right\rangle \right)$	$\left( \left( s_{1},0.0000\right) ,\left\langle 0.6389, 0.2289\right\rangle \right)$	$\left( \left( s_{3},-0.3333\right) ,\left\langle 0.5203 , 0.5809 \right\rangle \right)$	$\left( \left( s_{2},-0.3333\right) ,\left\langle 0.4393 ,0.6952 \right\rangle \right)$
${\mathcal {O}}_5$	$\left( \left( s_{5},0.3333\right) ,\left\langle 0.7726, 0.4000 \right\rangle \right)$	$\left( \left( s_{4},0.3333\right) ,\left\langle 0.3405, 0.2289 \right\rangle \right)$	$\left( \left( s_{2},-0.3333\right) ,\left\langle 0.7726, 0.3302\right\rangle \right)$	$\left( \left( s_{5},0.3333\right) ,\left\langle 0.6000, 0.5000\right\rangle \right)$
${\mathcal {O}}_6$	$\left( \left( s_{3},0.3333\right) ,\left\langle 0.4720,0.2621 \right\rangle \right)$	$\left( \left( s_{3},-0.3333\right) ,\left\langle 0.6000,0.4309 \right\rangle \right)$	$\left( \left( s_{3},0.0000\right) ,\left\langle 0.5203, 0.6649 \right\rangle \right)$	$\left( \left( s_{1},0.0000\right) ,\left\langle 0.4736,0.4762 \right\rangle \right)$
${\mathcal {O}}_7$	$\left( \left( s_{4},0.0000\right) ,\left\langle 0.5388,0.4642 \right\rangle \right)$	$\left( \left( s_{5},0.3333\right) ,\left\langle 0.7172,0.7652 \right\rangle \right)$	$\left( \left( s_{5},0.0000\right) ,\left\langle 0.5159,0.6000 \right\rangle \right)$	$\left( \left( s_{2},0.3333\right) ,\left\langle 0.4720,0.4309 \right\rangle \right)$

Step 3: Based on maximizing deviation model^19^, criteria weights are determined as:

$$\begin{aligned} \varpi _1=0.2353, \varpi _2=0.2727, \varpi _3=0.1912, \varpi _4=0.3008. \end{aligned}$$

Step 4: Determine the correlation coefficients of the q-ROPFLSs ${\mathcal {O}}_i(i=1,2,...,7)$ utilizing Eq. (14) and the weight vector $\varpi = \left( 0.2353, 0.2727, 0.1912, 0.3008\right)$. The resulting correlation matrix is:

$$\begin{aligned} C=\left( \begin{array}{ccccccc} 1 &{} 0.1224 &{} 0.7030 &{} 0.4552 &{} 0.4661 &{} 0.6978 &{} 0.6587 \\ 0.1224 &{} 1 &{} 0.6220 &{} 0.4388 &{} 0.5499 &{} 0.3357 &{} 0.3357 \\ 0.7030 &{} 0.6220 &{} 1 &{} 0.8206 &{} 0.6848 &{} 0.7002 &{} 0.8005 \\ 0.5075 &{} 0.4388 &{} 0.8206 &{} 1 &{} 0.7634 &{} 0.6469 &{} 0.4324 \\ 0.4661 &{} 0.5499 &{} 0.6848 &{} 0.7634 &{} 1 &{} 0.4469 &{} 0.2965 \\ 0.6978 &{} 0.3357 &{} 0.7002 &{} 0.6469 &{} 0.4469 &{} 1 &{} 0.7590 \\ 0.6587 &{} 0.4032 &{} 0.8612 &{} 0.4324 &{} 0.2965 &{} 0.7590 &{} 1 \\ \end{array} \right) . \end{aligned}$$

Step 5: Find out the equivalent correlation matrix:

$$\begin{aligned}{} & {} C^2=C\circ C=\left( \begin{array}{ccccccc} 1 &{} 0.6220 &{} 0.7030 &{} 0.7030 &{} 0.6848 &{} 0.7002 &{} 0.7030 \\ 0.6220 &{} 1 &{} 0.6220 &{} 0.6220 &{} 0.6220 &{} 0.6220 &{} 0.6220 \\ 0.7030 &{} 0.6220 &{} 1 &{} 0.8206 &{} 0.7634 &{} 0.7590 &{} 0.8005 \\ 0.7030 &{} 0.6220 &{} 0.8206 &{} 1 &{} 0.7634 &{} 0.7002 &{} 0.8005 \\ 0.6848 &{} 0.6220 &{} 0.7634 &{} 0.7634 &{} 1 &{} 0.6848 &{} 0.6848 \\ 0.7002 &{} 0.6220 &{} 0.7590 &{} 0.7002 &{} 0.6848 &{} 1 &{} 0.7590 \\ 0.7030 &{} 0.6220 &{} 0.8612 &{} 0.8206 &{} 0.6848 &{} 0.7590 &{} 1 \\ \end{array} \right) \\{} & {} \quad C^4=C^2\circ C^2=\left( \begin{array}{ccccccc} 1 &{} 0.6220 &{} 0.7030 &{} 0.7030 &{} 0.7030 &{} 0.7030 &{} 0.7030 \\ 0.6220 &{} 1 &{} 0.6220 &{} 0.6220 &{} 0.6220 &{} 0.6220 &{} 0.6220 \\ 0.7030 &{} 0.6220 &{} 1 &{} 0.8206 &{} 0.7634 &{} 0.7590 &{} 0.8005 \\ 0.7030 &{} 0.6220 &{} 0.8206 &{} 1 &{} 0.7634 &{} 0.7590 &{} 0.8005 \\ 0.7030 &{} 0.6220 &{} 0.7634 &{} 0.7634 &{} 1 &{} 0.7590 &{} 0.7634 \\ 0.7030 &{} 0.6220 &{} 0.7590 &{} 0.7590 &{} 0.7590 &{} 1 &{} 0.7590 \\ 0.7030 &{} 0.6220 &{} 0.8612 &{} 0.8206 &{} 0.7634 &{} 0.7590 &{} 1 \\ \end{array} \right) \\{} & {} \quad C^8=C^4\circ C^4=\left( \begin{array}{ccccccc} 1 &{} 0.6220 &{} 0.7030 &{} 0.7030 &{} 0.7030 &{} 0.7030 &{} 0.7030 \\ 0.6220 &{} 1 &{} 0.6220 &{} 0.6220 &{} 0.6220 &{} 0.6220 &{} 0.6220 \\ 0.7030 &{} 0.6220 &{} 1 &{} 0.8206 &{} 0.7634 &{} 0.7590 &{} 0.8005 \\ 0.7030 &{} 0.6220 &{} 0.8206 &{} 1 &{} 0.7634 &{} 0.7590 &{} 0.8005 \\ 0.7030 &{} 0.6220 &{} 0.7634 &{} 0.7634 &{} 1 &{} 0.7590 &{} 0.7634 \\ 0.7030 &{} 0.6220 &{} 0.7590 &{} 0.7590 &{} 0.7590 &{} 1 &{} 0.7590 \\ 0.7030 &{} 0.6220 &{} 0.8612 &{} 0.8206 &{} 0.7634 &{} 0.7590 &{} 1 \\ \end{array} \right) \end{aligned}$$

Hence, $C^4$ is an equivalent correlation matrix.

Step 6: In the light of Eq. (19) to generate a $\zeta$-cutting matrix $C_{\zeta }=\left( \zeta \rho _{ij} \right) _{7 \times 7}$ from which all plausible classes of the softwares ${\mathcal {O}}_i(i = 1,2,..., 7)$ can be derived:

(i) If $0\le \zeta \le 0.6220$, then ${\mathcal {O}}_i(i = 1,2,..., 7)$ are of the same type:
  $$\begin{aligned} \left\{ {\mathcal {O}}_1,{\mathcal {O}}_2,{\mathcal {O}}_3,{\mathcal {O}}_4,{\mathcal {O}}_5, {\mathcal {O}}_6,{\mathcal {O}}_7 \right\} . \end{aligned}$$
(ii) If $0.6220< \zeta \le 0.7030$, then ${\mathcal {O}}_i(i = 1,2,..., 7)$ are categorized into two types:
  $$\begin{aligned} \left\{ {\mathcal {O}}_2\right\} , \left\{ {\mathcal {O}}_1,{\mathcal {O}}_3,{\mathcal {O}}_4,{\mathcal {O}}_5,{\mathcal {O}}_6,{\mathcal {O}}_7 \right\} . \end{aligned}$$
(iii) If $0.7030< \zeta \le 0.7590$, then ${\mathcal {O}}_i(i = 1,2,..., 7)$ are categorized into three types:
  $$\begin{aligned} \left\{ {\mathcal {O}}_1\right\} ,\left\{ {\mathcal {O}}_2\right\} , \left\{ {\mathcal {O}}_3,{\mathcal {O}}_4,{\mathcal {O}}_5,{\mathcal {O}}_6,{\mathcal {O}}_7 \right\} . \end{aligned}$$
(iv) If $0.7590< \zeta \le 0.7634$, then ${\mathcal {O}}_i(i = 1,2,..., 7)$ are categorized into four types:
  $$\begin{aligned} \left\{ {\mathcal {O}}_1\right\} ,\left\{ {\mathcal {O}}_2\right\} , \left\{ {\mathcal {O}}_3,{\mathcal {O}}_4,{\mathcal {O}}_5,{\mathcal {O}}_7 \right\} ,\left\{ {\mathcal {O}}_6\right\} . \end{aligned}$$
(v) If $0.7634< \zeta \le 0.8005$, then ${\mathcal {O}}_i(i = 1,2,..., 7)$ are categorized into five types:
  $$\begin{aligned} \left\{ {\mathcal {O}}_1\right\} ,\left\{ {\mathcal {O}}_2\right\} , \left\{ {\mathcal {O}}_3,{\mathcal {O}}_4, {\mathcal {O}}_7 \right\} ,\left\{ {\mathcal {O}}_5\right\} ,\left\{ {\mathcal {O}}_6\right\} . \end{aligned}$$
(vi) If $0.8005< \zeta \le 0.8206$, then ${\mathcal {O}}_i(i = 1,2,..., 7)$ are categorized into six types:
  $$\begin{aligned} \left\{ {\mathcal {O}}_1\right\} ,\left\{ {\mathcal {O}}_2\right\} , \left\{ {\mathcal {O}}_3,{\mathcal {O}}_4 \right\} ,\left\{ {\mathcal {O}}_5\right\} ,\left\{ {\mathcal {O}}_6\right\} ,\left\{ {\mathcal {O}}_7\right\} . \end{aligned}$$
(vii) If $0.8206< \zeta \le 1$, then ${\mathcal {O}}_i(i = 1,2,..., 7)$ are categorized into seven types:
  $$\begin{aligned} \left\{ {\mathcal {O}}_1\right\} ,\left\{ {\mathcal {O}}_2\right\} , \left\{ {\mathcal {O}}_3, \right\} ,\left\{ {\mathcal {O}}_4\right\} ,\left\{ {\mathcal {O}}_5\right\} ,\left\{ {\mathcal {O}}_6\right\} ,\left\{ {\mathcal {O}}_7\right\} . \end{aligned}$$

#### How to choose the optimal value of $\zeta$ ?

The framed method categorizes the q-ROPTLSs under the provided confidence levels by utilizing the $\zeta$-cutting matrix of the corresponding correlation matrix. Given that confidence levels have a close relationship with the elements of equivalent correlation matrices, people can properly specify the confidence levels in practical applications based on the elements of the equivalent correlation matrices and the actual situations, and thus the framed algorithm has desirable flexibility and practicality.

### Parameter analysis

This section discusses option clustering when the parameter *q* values fluctuate. In the deployed algorithm, several values of *q* are used for this, as demonstrated below.

**Case 1:** Clustering analysis using $q=3:$

In the preceding section, the clustering method for q=3 has already been executed. Thus, we proceed to the subsequent cases.

Using the obtained weight vector $\varpi = \left( 0.2353, 0.2727, 0.1912, 0.3008\right)$ ( for q=3), and following the Steps 4-6 of clustering algorithm outlined in the preceding section, the case-by-case computations are performed as follows.

**Case 2:** Clustering analysis using $q=5:$

Step 4: Based on Eq. (14) the correlation matrix of the q-ROPFLSs ${\mathcal {O}}_i(i=1,2,...,7)$ is obtained as:

$$\begin{aligned} C=\left( \begin{array}{ccccccc} 1 &{} 0.02946 &{} 0.7173 &{} 0.3717 &{} 0.3520 &{} 0.5862 &{} 0.5823 \\ 0.02946 &{} 1 &{} 0.5425 &{} 0.4486 &{} 0.3307 &{} 0.1523 &{} 0.2375 \\ 0.7173 &{} 0.5425 &{} 1 &{} 0.7221 &{} 0.4351 &{} 0.5846 &{} 0.7997 \\ 0.4198 &{} 0.4486 &{} 0.7221 &{} 1 &{} 0.5964 &{} 0.5599 &{} 0.2917 \\ 0.3520 &{} 0.3307 &{} 0.4351 &{} 0.5964 &{} 1 &{} 0.2579 &{} 0.1320 \\ 0.5862 &{} 0.1523 &{} 0.5846 &{} 0.5599 &{} 0.2579 &{} 1 &{} 0.5532 \\ 0.5823 &{} 0.2343 &{} 0.8212 &{} 0.2917 &{} 0.1320 &{} 0.5532 &{} 1 \\ \end{array} \right) . \end{aligned}$$

Step 5: Determine the equivalent correlation matrix:

$$\begin{aligned}{} & {} C^2=C\circ C=\left( \begin{array}{ccccccc} 1 &{} 0.5425 &{} 0.7173 &{} 0.7173 &{} 0.4351 &{} 0.5862 &{} 0.7173 \\ 0.5425 &{} 1 &{} 0.5425 &{} 0.5425 &{} 0.4486 &{} 0.5425 &{} 0.5425 \\ 0.7173 &{} 0.5425 &{} 1 &{} 0.7221 &{} 0.5964 &{} 0.5862 &{} 0.7997 \\ 0.7173 &{} 0.5425 &{} 0.7221 &{} 1 &{} 0.5964 &{} 0.5846 &{} 0.7221 \\ 0.4351 &{} 0.4486 &{} 0.5964 &{} 0.5964 &{} 1 &{} 0.5599 &{} 0.4351 \\ 0.5862 &{} 0.5425 &{} 0.5862 &{} 0.5846 &{} 0.5599 &{} 1 &{} 0.5846 \\ 0.7173 &{} 0.5425 &{} 0.8212 &{} 0.7221 &{} 0.4351 &{} 0.5846 &{} 1 \\ \end{array} \right) \\{} & {} \quad C^4=C^2\circ C^2=\left( \begin{array}{ccccccc} 1 &{} 0.5425 &{} 0.7173 &{} 0.7173 &{} 0.5964 &{} 0.5862 &{} 0.7173 \\ 0.5425 &{} 1 &{} 0.5425 &{} 0.5425 &{} 0.5425 &{} 0.5425 &{} 0.5425 \\ 0.7173 &{} 0.5425 &{} 1 &{} 0.7221 &{} 0.5964 &{} 0.5862 &{} 0.7997 \\ 0.7173 &{} 0.5425 &{} 0.7221 &{} 1 &{} 0.5964 &{} 0.5862 &{} 0.7221 \\ 0.5964 &{} 0.5425 &{} 0.5964 &{} 0.5964 &{} 1 &{} 0.5862 &{} 0.5964 \\ 0.5862 &{} 0.5425 &{} 0.5862 &{} 0.5862 &{} 0.5862 &{} 1 &{} 0.5862 \\ 0.7173 &{} 0.5425 &{} 0.8212 &{} 0.7221 &{} 0.5964 &{} 0.5862 &{} 1 \\ \end{array} \right) \\{} & {} \quad C^8=C^4\circ C^4=\left( \begin{array}{ccccccc} 1 &{} 0.5425 &{} 0.7173 &{} 0.7173 &{} 0.5964 &{} 0.5862 &{} 0.7173 \\ 0.5425 &{} 1 &{} 0.5425 &{} 0.5425 &{} 0.5425 &{} 0.5425 &{} 0.5425 \\ 0.7173 &{} 0.5425 &{} 1 &{} 0.7221 &{} 0.5964 &{} 0.5862 &{} 0.7997 \\ 0.7173 &{} 0.5425 &{} 0.7221 &{} 1 &{} 0.5964 &{} 0.5862 &{} 0.7221 \\ 0.5964 &{} 0.5425 &{} 0.5964 &{} 0.5964 &{} 1 &{} 0.5862 &{} 0.5964 \\ 0.5862 &{} 0.5425 &{} 0.5862 &{} 0.5862 &{} 0.5862 &{} 1 &{} 0.5862 \\ 0.7173 &{} 0.5425 &{} 0.8212 &{} 0.7221 &{} 0.5964 &{} 0.5862 &{} 1 \\ \end{array} \right) . \end{aligned}$$

Step 6: In the light of Eq. (19) to generate a $\zeta$-cutting matrix $C_{\zeta }=\left( \zeta \rho _{ij} \right) _{7 \times 7}$ from which all plausible classes of the softwares ${\mathcal {O}}_i(i = 1,2,..., 7)$ can be derived:

(i) If $0\le \zeta \le 0.5425$, then ${\mathcal {O}}_i(i = 1,2,..., 7)$ are of the same type:
  $$\begin{aligned} \left\{ {\mathcal {O}}_1,{\mathcal {O}}_2,{\mathcal {O}}_3,{\mathcal {O}}_4,{\mathcal {O}}_5,{\mathcal {O}}_6,{\mathcal {O}}_7 \right\} . \end{aligned}$$
(ii) If $0.5425< \zeta \le 0.5862$, then ${\mathcal {O}}_i(i = 1,2,..., 7)$ are categorized into two types:
  $$\begin{aligned} \left\{ {\mathcal {O}}_2\right\} , \left\{ {\mathcal {O}}_1,{\mathcal {O}}_3,{\mathcal {O}}_4,{\mathcal {O}}_5,{\mathcal {O}}_6,{\mathcal {O}}_7 \right\} . \end{aligned}$$
(iii) If $0.5862< \zeta \le 0.5964$, then ${\mathcal {O}}_i(i = 1,2,..., 7)$ are categorized into three types:
  $$\begin{aligned} \left\{ {\mathcal {O}}_2\right\} ,\left\{ {\mathcal {O}}_6\right\} , \left\{ {\mathcal {O}}_1,{\mathcal {O}}_3,{\mathcal {O}}_4,{\mathcal {O}}_5,{\mathcal {O}}_7 \right\} . \end{aligned}$$
(iv) If $0.5964< \zeta \le 0.7173$, then ${\mathcal {O}}_i(i = 1,2,..., 7)$ are categorized into four types:
  $$\begin{aligned} \left\{ {\mathcal {O}}_1,{\mathcal {O}}_3,{\mathcal {O}}_4,{\mathcal {O}}_7\right\} ,\left\{ {\mathcal {O}}_2\right\} ,\left\{ {\mathcal {O}}_5\right\} ,\left\{ {\mathcal {O}}_6\right\} . \end{aligned}$$
(v) If $0.7173< \zeta \le 0.7221$, then ${\mathcal {O}}_i(i = 1,2,..., 7)$ are categorized into five types:
  $$\begin{aligned} \left\{ {\mathcal {O}}_1\right\} ,\left\{ {\mathcal {O}}_2\right\} , \left\{ {\mathcal {O}}_3,{\mathcal {O}}_4, {\mathcal {O}}_7 \right\} ,\left\{ {\mathcal {O}}_5\right\} ,\left\{ {\mathcal {O}}_6\right\} . \end{aligned}$$
(vi) If $0.7221< \zeta \le 0.7997$, then ${\mathcal {O}}_i(i = 1,2,..., 7)$ are categorized into six types:
  $$\begin{aligned} \left\{ {\mathcal {O}}_1\right\} ,\left\{ {\mathcal {O}}_2\right\} , \left\{ {\mathcal {O}}_3,{\mathcal {O}}_7 \right\} ,\left\{ {\mathcal {O}}_4\right\} ,\left\{ {\mathcal {O}}_5\right\} ,\left\{ {\mathcal {O}}_6\right\} . \end{aligned}$$
(vii) If $0.7997< \zeta \le 1$, then ${\mathcal {O}}_i(i = 1,2,..., 7)$ are categorized into seven types:
  $$\begin{aligned} \left\{ {\mathcal {O}}_1\right\} ,\left\{ {\mathcal {O}}_2\right\} , \left\{ {\mathcal {O}}_3\right\} ,\left\{ {\mathcal {O}}_4 \right\} ,\left\{ {\mathcal {O}}_5\right\} ,\left\{ {\mathcal {O}}_6\right\} ,\left\{ {\mathcal {O}}_7\right\} . \end{aligned}$$

**Case 3:** Clustering analysis using $q=7:$

Step 4: According to Eq. (14) the correlation matrix of the q-ROPFLSs ${\mathcal {O}}_i(i=1,2,...,7)$ is obtained as:

$$\begin{aligned}C=\left( \begin{array}{ccccccc} 1 &{} 0.007736 &{} 0.7596 &{} 0.3120 &{} 0.2729 &{} 0.4863 &{} 0.5289 \\ 0.007736 &{} 1 &{} 0.4479 &{} 0.4693 &{} 0.1920 &{} 0.06952 &{} 0.1461 \\ 0.7596 &{} 0.4479 &{} 1 &{} 0.6485 &{} 0.2597 &{} 0.4794 &{} 0.7857 \\ 0.3490 &{} 0.4693 &{} 0.6485 &{} 1 &{} 0.4026 &{} 0.4523 &{} 0.2032 \\ 0.2729 &{} 0.1920 &{} 0.2597 &{} 0.4026 &{} 1 &{} 0.1436 &{} 0.06283 \\ 0.4863 &{} 0.06952 &{} 0.4794 &{} 0.4523 &{} 0.1436 &{} 1 &{} 0.3927 \\ 0.5289 &{} 0.1453 &{} 0.7920 &{} 0.2032 &{} 0.06283 &{} 0.3927 &{} 1 \\ \end{array} \right) . \end{aligned}$$

Step 5: Calculate the equivalent correlation matrix:

$$\begin{aligned}{} & {} C^2=C\circ C=\left( \begin{array}{ccccccc} 1 &{} 0.4479 &{} 0.7596 &{} 0.6485 &{} 0.3120 &{} 0.4863 &{} 0.7596 \\ 0.4479 &{} 1 &{} 0.4693 &{} 0.4693 &{} 0.4026 &{} 0.4523 &{} 0.4479 \\ 0.7596 &{} 0.4693 &{} 1 &{} 0.6485 &{} 0.4026 &{} 0.4863 &{} 0.7857 \\ 0.6485 &{} 0.4693 &{} 0.6485 &{} 1 &{} 0.4026 &{} 0.4794 &{} 0.6485 \\ 0.3490 &{} 0.4026 &{} 0.4026 &{} 0.4026 &{} 1 &{} 0.4026 &{} 0.2729 \\ 0.4863 &{} 0.4523 &{} 0.4863 &{} 0.4794 &{} 0.4026 &{} 1 &{} 0.4863 \\ 0.7596 &{} 0.4479 &{} 0.7920 &{} 0.6485 &{} 0.2729 &{} 0.4863 &{} 1 \\ \end{array} \right) \\{} & {} \quad C^4=C^2\circ C^2=\left( \begin{array}{ccccccc} 1 &{} 0.4693 &{} 0.7596 &{} 0.6485 &{} 0.4026 &{} 0.4863 &{} 0.7596 \\ 0.4693 &{} 1 &{} 0.4693 &{} 0.4693 &{} 0.4026 &{} 0.4693 &{} 0.4693 \\ 0.7596 &{} 0.4693 &{} 1 &{} 0.6485 &{} 0.4026 &{} 0.4863 &{} 0.7857 \\ 0.6485 &{} 0.4693 &{} 0.6485 &{} 1 &{} 0.4026 &{} 0.4863 &{} 0.6485 \\ 0.4026 &{} 0.4026 &{} 0.4026 &{} 0.4026 &{} 1 &{} 0.4026 &{} 0.4026 \\ 0.4863 &{} 0.4693 &{} 0.4863 &{} 0.4863 &{} 0.4026 &{} 1 &{} 0.4863 \\ 0.7596 &{} 0.4693 &{} 0.7920 &{} 0.6485 &{} 0.4026 &{} 0.4863 &{} 1 \\ \end{array} \right) \\{} & {} \quad C^8=C^4\circ C^4=\left( \begin{array}{ccccccc} 1 &{} 0.4693 &{} 0.7596 &{} 0.6485 &{} 0.4026 &{} 0.4863 &{} 0.7596 \\ 0.4693 &{} 1 &{} 0.4693 &{} 0.4693 &{} 0.4026 &{} 0.4693 &{} 0.4693 \\ 0.7596 &{} 0.4693 &{} 1 &{} 0.6485 &{} 0.4026 &{} 0.4863 &{} 0.7857 \\ 0.6485 &{} 0.4693 &{} 0.6485 &{} 1 &{} 0.4026 &{} 0.4863 &{} 0.6485 \\ 0.4026 &{} 0.4026 &{} 0.4026 &{} 0.4026 &{} 1 &{} 0.4026 &{} 0.4026 \\ 0.4863 &{} 0.4693 &{} 0.4863 &{} 0.4863 &{} 0.4026 &{} 1 &{} 0.4863 \\ 0.7596 &{} 0.4693 &{} 0.7920 &{} 0.6485 &{} 0.4026 &{} 0.4863 &{} 1 \\ \end{array} \right) . \end{aligned}$$

Step 6: In the light of Eq. (19) to generate a $\zeta$-cutting matrix $C_{\zeta }=\left( \zeta \rho _{ij} \right) _{7 \times 7}$ from which all plausible classes of the softwares ${\mathcal {O}}_i(i = 1,2,..., 7)$ can be derived:

(i) If $0\le \zeta \le 0.4026$, then ${\mathcal {O}}_i(i = 1,2,..., 7)$ are of the same type:
  $$\begin{aligned} \left\{ {\mathcal {O}}_1,{\mathcal {O}}_2,{\mathcal {O}}_3,{\mathcal {O}}_4,{\mathcal {O}}_5, {\mathcal {O}}_6,{\mathcal {O}}_7 \right\} . \end{aligned}$$
(ii) If $0.4026< \zeta \le 0.4693$, then ${\mathcal {O}}_i(i = 1,2,..., 7)$ are categorized into two types:
  $$\begin{aligned} \left\{ {\mathcal {O}}_1,{\mathcal {O}}_2,{\mathcal {O}}_3,{\mathcal {O}}_4,{\mathcal {O}}_6,{\mathcal {O}}_7 \right\} ,\left\{ {\mathcal {O}}_5\right\} . \end{aligned}$$
(iii) If $0.4693< \zeta \le 0.4863$, then ${\mathcal {O}}_i(i = 1,2,..., 7)$ are categorized into three types:
  $$\begin{aligned} \left\{ {\mathcal {O}}_1,{\mathcal {O}}_3,{\mathcal {O}}_4,{\mathcal {O}}_6,{\mathcal {O}}_7 \right\} ,\left\{ {\mathcal {O}}_2\right\} ,\left\{ {\mathcal {O}}_5\right\} . \end{aligned}$$
(iv) If $0.4863< \zeta \le 0.6485$, then ${\mathcal {O}}_i(i = 1,2,..., 7)$ are categorized into four types:
  $$\begin{aligned} \left\{ {\mathcal {O}}_2\right\} , \left\{ {\mathcal {O}}_1,{\mathcal {O}}_3,{\mathcal {O}}_4,{\mathcal {O}}_7 \right\} ,\left\{ {\mathcal {O}}_5\right\} , \left\{ {\mathcal {O}}_6\right\} . \end{aligned}$$
(v) If $0.6485< \zeta \le 0.7596$, then ${\mathcal {O}}_i(i = 1,2,..., 7)$ are categorized into five types:
  $$\begin{aligned} \left\{ {\mathcal {O}}_1,{\mathcal {O}}_3,{\mathcal {O}}_7\right\} ,\left\{ {\mathcal {O}}_2\right\} , \left\{ ,{\mathcal {O}}_4, \right\} ,\left\{ {\mathcal {O}}_5\right\} ,\left\{ {\mathcal {O}}_6\right\} . \end{aligned}$$
(vi) If $0.7596< \zeta \le 0.7857$, then ${\mathcal {O}}_i(i = 1,2,..., 7)$ are categorized into six types:
  $$\begin{aligned} \left\{ {\mathcal {O}}_1\right\} ,\left\{ {\mathcal {O}}_2\right\} , \left\{ {\mathcal {O}}_3, {\mathcal {O}}_7\right\} ,\left\{ {\mathcal {O}}_4 \right\} ,\left\{ {\mathcal {O}}_5\right\} ,\left\{ {\mathcal {O}}_6\right\} . \end{aligned}$$
(vii) If $0.7857< \zeta \le 1$, then ${\mathcal {O}}_i(i = 1,2,..., 7)$ are categorized into seven types:
  $$\begin{aligned} \left\{ {\mathcal {O}}_1\right\} ,\left\{ {\mathcal {O}}_2\right\} , \left\{ {\mathcal {O}}_3\right\} ,\left\{ {\mathcal {O}}_4 \right\} ,\left\{ {\mathcal {O}}_5\right\} ,\left\{ {\mathcal {O}}_6\right\} ,\left\{ {\mathcal {O}}_7\right\} . \end{aligned}$$

**Case 4:** Clustering analysis using $q=9:$

Step 4: On the basis of Eq. (14) the correlation matrix of the q-ROPFLSs ${\mathcal {O}}_i(i=1,2,...,7)$ is derived as:

$$\begin{aligned} C=\left( \begin{array}{ccccccc} 1 &{} 0.002137 &{} 0.8024 &{} 0.2632 &{} 0.2109 &{} 0.4015 &{} 0.4811 \\ 0.002137 &{} 1 &{} 0.3573 &{} 0.4621 &{} 0.1118 &{} 0.03242 &{} 0.09298 \\ 0.8024 &{} 0.3573 &{} 1 &{} 0.5874 &{} 0.1567 &{} 0.3933 &{} 0.7556 \\ 0.2850 &{} 0.4621 &{} 0.5874 &{} 1 &{} 0.2425 &{} 0.3389 &{} 0.1448 \\ 0.2109 &{} 0.1118 &{} 0.1567 &{} 0.2425 &{} 1 &{} 0.07925 &{} 0.03088 \\ 0.4015 &{} 0.03242 &{} 0.3933 &{} 0.3389 &{} 0.07925 &{} 1 &{} 0.2778 \\ 0.4811 &{} 0.09277 &{} 0.7573 &{} 0.1448 &{} 0.03088 &{} 0.2778 &{} 1 \\ \end{array} \right) . \end{aligned}$$

Step 5: Work out the equivalent correlation matrix:

$$\begin{aligned}{} & {} C^2=C\circ C=\left( \begin{array}{ccccccc} 1 &{} 0.3573 &{} 0.8024 &{} 0.5874 &{} 0.2425 &{} 0.4015 &{} 0.7556 \\ 0.3573 &{} 2 &{} 0.4621 &{} 0.4621 &{} 0.2425 &{} 0.3573 &{} 0.3573 \\ 0.8024 &{} 0.4621 &{} 1 &{} 0.5874 &{} 0.2425 &{} 0.4015 &{} 0.7556 \\ 0.5874 &{} 0.4621 &{} 0.5874 &{} 1 &{} 0.2425 &{} 0.3933 &{} 0.5874 \\ 0.5874 &{} 0.2425 &{} 0.2425 &{} 0.2425 &{} 1 &{} 0.2425 &{} 0.2109 \\ 0.4015 &{} 0.3573 &{} 0.4015 &{} 0.3933 &{} 0.2425 &{} 1 &{} 0.4015 \\ 0.7573 &{} 0.3573 &{} 0.7573 &{} 0.5874 &{} 0.2109 &{} 0.4015 &{} 1 \\ \end{array} \right) \\{} & {} \quad C^4=C^2\circ C^2=\left( \begin{array}{ccccccc} 1 &{} 0.4621 &{} 0.8024 &{} 0.8024 &{} 0.2425 &{} 0.4015 &{} 0.7556 \\ 0.4621 &{} 1 &{} 0.4621 &{} 0.4621 &{} 0.2425 &{} 0.4015 &{} 0.4621 \\ 0.8024 &{} 0.4621 &{} 1 &{} 0.5874 &{} 0.2425 &{} 0.4015 &{} 0.7556 \\ 0.5874 &{} 0.4621 &{} 0.5874 &{} 1 &{} 0.2425 &{} 0.4015 &{} 0.5874 \\ 0.5874 &{} 0.3573 &{} 0.5874 &{} 0.5874 &{} 1 &{} 0.4015 &{} 0.5874 \\ 0.4015 &{} 0.4015 &{} 0.4015 &{} 0.4015 &{} 0.2425 &{} 1 &{} 0.4015 \\ 0.7573 &{} 0.4621 &{} 0.7573 &{} 0.5874 &{} 0.2425 &{} 0.4015 &{} 1 \\ \end{array} \right) \\{} & {} \quad C^8=C^4\circ C^4=\left( \begin{array}{ccccccc} 1 &{} 0.4621 &{} 0.8024 &{} 0.8024 &{} 0.2425 &{} 0.4015 &{} 0.7556 \\ 0.4621 &{} 1 &{} 0.4621 &{} 0.4621 &{} 0.2425 &{} 0.4015 &{} 0.4621 \\ 0.8024 &{} 0.4621 &{} 1 &{} 0.8024 &{} 0.2425 &{} 0.4015 &{} 0.7556 \\ 0.5874 &{} 0.4621 &{} 0.5874 &{} 1 &{} 0.2425 &{} 0.4015 &{} 0.5874 \\ 0.5874 &{} 0.4621 &{} 0.5874 &{} 0.5874 &{} 1 &{} 0.4015 &{} 0.5874 \\ 0.4015 &{} 0.4015 &{} 0.4015 &{} 0.4015 &{} 0.2425 &{} 1 &{} 0.4015 \\ 0.7573 &{} 0.4621 &{} 0.7573 &{} 0.7573 &{} 0.2425 &{} 0.4015 &{} 1 \\ \end{array} \right) . \end{aligned}$$

Step 6: In the light of Eq. (19) to generate a $\zeta$-cutting matrix $C_{\zeta }=\left( \zeta \rho _{ij} \right) _{7 \times 7}$ from which all plausible classes of the softwares ${\mathcal {O}}_i(i = 1,2,..., 7)$ can be derived:

(i) If $0\le \zeta \le 0.2425$, then ${\mathcal {O}}_i(i = 1,2,..., 7)$ are of the same type:
  $$\begin{aligned} \left\{ {\mathcal {O}}_1,{\mathcal {O}}_2,{\mathcal {O}}_3,{\mathcal {O}}_4,{\mathcal {O}}_5, {\mathcal {O}}_6,{\mathcal {O}}_7 \right\} . \end{aligned}$$
(ii) If $0.2425< \zeta \le 0.4015$, then ${\mathcal {O}}_i(i = 1,2,..., 7)$ are categorized into two types:
  $$\begin{aligned} \left\{ {\mathcal {O}}_1,{\mathcal {O}}_2,{\mathcal {O}}_3,{\mathcal {O}}_4,{\mathcal {O}}_6,{\mathcal {O}}_7 \right\} ,\left\{ {\mathcal {O}}_5\right\} \end{aligned}$$
(iii) If $0.4015< \zeta \le 0.4621$, then ${\mathcal {O}}_i(i = 1,2,..., 7)$ are categorized into three types:
  $$\begin{aligned} \left\{ {\mathcal {O}}_1,{\mathcal {O}}_2,{\mathcal {O}}_3,{\mathcal {O}}_4,{\mathcal {O}}_7 \right\} ,\left\{ {\mathcal {O}}_5\right\} ,\left\{ {\mathcal {O}}_6\right\} . \end{aligned}$$
(iv) If $0.4621< \zeta \le 0.5874$, then ${\mathcal {O}}_i(i = 1,2,..., 7)$ are categorized into four types:
  $$\begin{aligned} \left\{ {\mathcal {O}}_1,{\mathcal {O}}_3,{\mathcal {O}}_4,{\mathcal {O}}_7 \right\} ,\left\{ {\mathcal {O}}_2\right\} ,\left\{ {\mathcal {O}}_5\right\} ,\left\{ {\mathcal {O}}_6\right\} . \end{aligned}$$
(v) If $0.5874< \zeta \le 0.7556$, then ${\mathcal {O}}_i(i = 1,2,..., 7)$ are categorized into five types:
  $$\begin{aligned} \left\{ {\mathcal {O}}_1,{\mathcal {O}}_3,{\mathcal {O}}_7\right\} ,\left\{ {\mathcal {O}}_2\right\} , \left\{ {\mathcal {O}}_4 \right\} ,\left\{ {\mathcal {O}}_5\right\} ,\left\{ {\mathcal {O}}_6\right\} . \end{aligned}$$
(vi) If $0.7556< \zeta \le 0.7573$, then ${\mathcal {O}}_i(i = 1,2,..., 7)$ are categorized into six types:
  $$\begin{aligned} \left\{ {\mathcal {O}}_1,{\mathcal {O}}_3\right\} ,\left\{ {\mathcal {O}}_2\right\} , \left\{ {\mathcal {O}}_4 \right\} ,\left\{ {\mathcal {O}}_5\right\} ,\left\{ {\mathcal {O}}_6\right\} ,\left\{ {\mathcal {O}}_7\right\} . \end{aligned}$$
(vii) If $0.7573< \zeta \le 1$, then ${\mathcal {O}}_i(i = 1,2,..., 7)$ are categorized into seven types:
  $$\begin{aligned} \left\{ {\mathcal {O}}_1\right\} ,\left\{ {\mathcal {O}}_2\right\} , \left\{ {\mathcal {O}}_3\right\} ,\left\{ {\mathcal {O}}_4 \right\} ,\left\{ {\mathcal {O}}_5\right\} ,\left\{ {\mathcal {O}}_6\right\} ,\left\{ {\mathcal {O}}_7\right\} . \end{aligned}$$

**Case 5:** Clustering analysis using $q=11:$

Step 4: In the light of Eq. (14) the correlation matrix of the q-ROPFLSs ${\mathcal {O}}_i(i=1,2,...,7)$ is calculated as:

$$\begin{aligned} C=\left( \begin{array}{ccccccc} 1 &{} 0.0006080 &{} 0.8409 &{} 0.2225 &{} 0.1623 &{} 0.3308 &{} 0.4358 \\ 0.0006080 &{} 1 &{} 0.2802 &{} 0.4347 &{} 0.06596 &{} 0.01542 &{} 0.05985 \\ 0.8409 &{} 0.2802 &{} 1 &{} 0.5302 &{} 0.09965 &{} 0.3239 &{} 0.7138 \\ 0.2332 &{} 0.4347 &{} 0.5302 &{} 1 &{} 0.1378 &{} 0.2438 &{} 0.1053 \\ 0.1623 &{} 0.06596 &{} 0.09965 &{} 0.1378 &{} 1 &{} 0.04374 &{} 0.01534 \\ 0.3308 &{} 0.01542 &{} 0.3239 &{} 0.2438 &{} 0.04374 &{} 1 &{} 0.1966 \\ 0.4358 &{} 0.05980 &{} 0.7142 &{} 0.1053 &{} 0.01534 &{} 0.1966 &{} 1 \\ \end{array} \right) . \end{aligned}$$

Step 5: Formulate the equivalent correlation matrix:

$$\begin{aligned}{} & {} C^2=C\circ C=\left( \begin{array}{ccccccc} 1 &{} 0.2802 &{} 0.8409 &{} 0.5302 &{} 0.1623 &{} 0.3308 &{} 0.7138 \\ 0.2802 &{} 1 &{} 0.4347 &{} 0.4347 &{} 0.1378 &{} 0.2802 &{} 0.2802 \\ 0.8409 &{} 0.4347 &{} 1 &{} 0.5302 &{} 0.1623 &{} 0.3308 &{} 0.7138 \\ 0.5302 &{} 0.4347 &{} 0.5302 &{} 1 &{} 0.1623 &{} 0.3239 &{} 0.5302 \\ 0.1623 &{} 0.1378 &{} 0.1623 &{} 0.1623 &{} 1 &{} 0.1623 &{} 0.1623 \\ 0.3308 &{} 0.2802 &{} 0.3308 &{} 0.3239 &{} 0.1623 &{} 1 &{} 0.3308 \\ 0.7142 &{} 0.2802 &{} 0.7142 &{} 0.5302 &{} 0.1623 &{} 0.3308 &{} 1 \\ \end{array} \right) \\{} & {} \quad C^4=C^2\circ C^2=\left( \begin{array}{ccccccc} 1 &{} 0.4347 &{} 0.8409 &{} 0.5302 &{} 0.1623 &{} 0.3308 &{} 0.7138 \\ 0.4347 &{} 1 &{} 0.4347 &{} 0.4347 &{} 0.1623 &{} 0.3308 &{} 0.4347 \\ 0.8409 &{} 0.4347 &{} 1 &{} 0.5302 &{} 0.1623 &{} 0.3308 &{} 0.7138 \\ 0.5302 &{} 0.4347 &{} 0.5302 &{} 1 &{} 0.1623 &{} 0.3308 &{} 0.5302 \\ 0.1623 &{} 0.1623 &{} 0.1623 &{} 0.1623 &{} 1 &{} 0.1623 &{} 0.1623 \\ 0.3308 &{} 0.3308 &{} 0.3308 &{} 0.3308 &{} 0.1623 &{} 1 &{} 0.3308 \\ 0.7142 &{} 0.4347 &{} 0.7142 &{} 0.5302 &{} 0.1623 &{} 0.3308 &{} 1 \\ \end{array} \right) \\{} & {} \quad C^8=C^4\circ C^4=\left( \begin{array}{ccccccc} 1 &{} 0.4347 &{} 0.8409 &{} 0.5302 &{} 0.1623 &{} 0.3308 &{} 0.7138 \\ 0.4347 &{} 1 &{} 0.4347 &{} 0.4347 &{} 0.1623 &{} 0.3308 &{} 0.4347 \\ 0.8409 &{} 0.4347 &{} 1 &{} 0.5302 &{} 0.1623 &{} 0.3308 &{} 0.7138 \\ 0.5302 &{} 0.4347 &{} 0.5302 &{} 1 &{} 0.1623 &{} 0.3308 &{} 0.5302 \\ 0.1623 &{} 0.1623 &{} 0.1623 &{} 0.1623 &{} 1 &{} 0.1623 &{} 0.1623 \\ 0.3308 &{} 0.3308 &{} 0.3308 &{} 0.3308 &{} 0.1623 &{} 1 &{} 0.3308 \\ 0.7142 &{} 0.4347 &{} 0.7142 &{} 0.5302 &{} 0.1623 &{} 0.3308 &{} 1 \\ \end{array} \right) . \end{aligned}$$

Step 6: In the light of Eq. (19) to generate a $\zeta$-cutting matrix $C_{\zeta }=\left( \zeta \rho _{ij} \right) _{7 \times 7}$ from which all plausible classes of the softwares ${\mathcal {O}}_i(i = 1,2,..., 7)$ can be derived:

(i) If $0\le \zeta \le 0.1623$, then ${\mathcal {O}}_i(i = 1,2,..., 7)$ are of the same type:
  $$\begin{aligned} \left\{ {\mathcal {O}}_1,{\mathcal {O}}_2,{\mathcal {O}}_3,{\mathcal {O}}_4,{\mathcal {O}}_5,{\mathcal {O}}_6,{\mathcal {O}}_7 \right\} . \end{aligned}$$
(ii) If $0.1623< \zeta \le 0.3308$, then ${\mathcal {O}}_i(i = 1,2,..., 7)$ are categorized into two types:
  $$\begin{aligned} \left\{ {\mathcal {O}}_1,{\mathcal {O}}_2,{\mathcal {O}}_3,{\mathcal {O}}_4,{\mathcal {O}}_6,{\mathcal {O}}_7 \right\} ,\left\{ {\mathcal {O}}_5\right\} . \end{aligned}$$
(iii) If $0.3308< \zeta \le 0.4347$, then ${\mathcal {O}}_i(i = 1,2,..., 7)$ are categorized into three types:
  $$\begin{aligned} \left\{ {\mathcal {O}}_1,{\mathcal {O}}_2,{\mathcal {O}}_3,{\mathcal {O}}_4,{\mathcal {O}}_7 \right\} ,\left\{ {\mathcal {O}}_5\right\} ,\left\{ {\mathcal {O}}_6\right\} . \end{aligned}$$
(iv) If $0.4347< \zeta \le 0.5302$, then ${\mathcal {O}}_i(i = 1,2,..., 7)$ are categorized into four types:
  $$\begin{aligned} \left\{ {\mathcal {O}}_1,{\mathcal {O}}_3,{\mathcal {O}}_4, {\mathcal {O}}_7 \right\} ,\left\{ {\mathcal {O}}_2\right\} ,\left\{ {\mathcal {O}}_5\right\} , \left\{ {\mathcal {O}}_6\right\} . \end{aligned}$$
(v) If $0.5302< \zeta \le 0.7138$, then ${\mathcal {O}}_i(i = 1,2,..., 7)$ are categorized into five types:
  $$\begin{aligned} \left\{ {\mathcal {O}}_1,{\mathcal {O}}_3,{\mathcal {O}}_7\right\} ,\left\{ {\mathcal {O}}_2\right\} , \left\{ {\mathcal {O}}_4 \right\} ,\left\{ {\mathcal {O}}_5\right\} ,\left\{ {\mathcal {O}}_6\right\} . \end{aligned}$$
(vi) If $0.7138< \zeta \le 0.7142$, then ${\mathcal {O}}_i(i = 1,2,..., 7)$ are categorized into six types:
  $$\begin{aligned} \left\{ {\mathcal {O}}_1\right\} ,\left\{ {\mathcal {O}}_2\right\} , \left\{ {\mathcal {O}}_3,{\mathcal {O}}_7 \right\} ,\left\{ {\mathcal {O}}_4\right\} ,\left\{ {\mathcal {O}}_5\right\} ,\left\{ {\mathcal {O}}_6\right\} . \end{aligned}$$
(vii) If $0.7142< \zeta \le 1$, then ${\mathcal {O}}_i(i = 1,2,..., 7)$ are categorized into seven types:
  $$\begin{aligned} \left\{ {\mathcal {O}}_1\right\} ,\left\{ {\mathcal {O}}_2\right\} , \left\{ {\mathcal {O}}_3\right\} ,\left\{ {\mathcal {O}}_4 \right\} ,\left\{ {\mathcal {O}}_5\right\} ,\left\{ {\mathcal {O}}_6\right\} ,\left\{ {\mathcal {O}}_7\right\} . \end{aligned}$$

**Case 6:** Clustering analysis using $q=15:$

Step 4: Utilizing Eq. (14) the correlation matrix of the q-ROPFLSs ${\mathcal {O}}_i(i=1,2,...,7)$ is figure out as:

$$\begin{aligned} C=\left( \begin{array}{ccccccc} 1 &{} 0.00005161 &{} 0.9012 &{} 0.1593 &{} 0.09632 &{} 0.2232 &{} 0.3520 \\ 0.00005161 &{} 1 &{} 0.1679 &{} 0.3629 &{} 0.02365 &{} 0.003627 &{} 0.02483 \\ 0.9012 &{} 0.1679 &{} 1 &{} 0.4231 &{} 0.04941 &{} 0.2202 &{} 0.6104 \\ 0.1614 &{} 0.3629 &{} 0.4231 &{} 1 &{} 0.04221 &{} 0.1210 &{} 0.05783 \\ 0.09632 &{} 0.02365 &{} 0.04941 &{} 0.04221 &{} 1 &{} 0.01352 &{} 0.003797 \\ 0.2232 &{} 0.003627 &{} 0.2202 &{} 0.1210 &{} 0.01352 &{} 1 &{} 0.09861 \\ 0.3520 &{} 0.02482 &{} 0.6105 &{} 0.05783 &{} 0.003797 &{} 0.09861 &{} 1 \\ \end{array} \right) . \end{aligned}$$

Step 5: Compute the equivalent correlation matrix:

$$\begin{aligned}{} & {} C^2=C\circ C=\left( \begin{array}{ccccccc} 1 &{} 0.1679 &{} 0.9012 &{} 0.4231 &{} 0.09632 &{} 0.2232 &{} 0.6104 \\ 0.1679 &{} 1 &{} 0.3629 &{} 0.3629 &{} 0.04941 &{} 0.1679 &{} 0.1679 \\ 0.9012 &{} 0.3629 &{} 1 &{} 0.4231 &{} 0.09632 &{} 0.2232 &{} 0.6104 \\ 0.4231 &{} 0.3629 &{} 0.4231 &{} 1 &{} 0.09632 &{} 0.2202 &{} 0.4231 \\ 0.09632 &{} 0.04941 &{} 0.09632 &{} 0.09632 &{} 1 &{} 0.09632 &{} 0.09632 \\ 0.2232 &{} 0.1679 &{} 0.2232 &{} 0.2202 &{} 0.09632 &{} 1 &{} 0.2232 \\ 0.6105 &{} 0.1679 &{} 0.6105 &{} 0.4231 &{} 0.09632 &{} 0.2232 &{} 1 \\ \end{array} \right) \\{} & {} \quad C^4=C^2\circ C^2=\left( \begin{array}{ccccccc} 1 &{} 0.3629 &{} 0.9012 &{} 0.4231 &{} 0.09632 &{} 0.2232 &{} 0.6104 \\ 0.3629 &{} 1 &{} 0.3629 &{} 0.3629 &{} 0.09632 &{} 0.2232 &{} 0.3629 \\ 0.9012 &{} 0.3629 &{} 1 &{} 0.4231 &{} 0.09632 &{} 0.2232 &{} 0.6104 \\ 0.4231 &{} 0.3629 &{} 0.4231 &{} 1 &{} 0.09632 &{} 0.2232 &{} 0.4231 \\ 0.09632 &{} 0.09632 &{} 0.09632 &{} 0.09632 &{} 1 &{} 0.09632 &{} 0.09632 \\ 0.2232 &{} 0.2232 &{} 0.2232 &{} 0.2232 &{} 0.09632 &{} 1 &{} 0.2232 \\ 0.6105 &{} 0.3629 &{} 0.6105 &{} 0.4231 &{} 0.09632 &{} 0.2232 &{} 1 \\ \end{array} \right) \\{} & {} \quad C^8=C^4\circ C^4=\left( \begin{array}{ccccccc} 1 &{} 0.3629 &{} 0.9012 &{} 0.4231 &{} 0.09632 &{} 0.2232 &{} 0.6104 \\ 0.3629 &{} 1 &{} 0.3629 &{} 0.3629 &{} 0.09632 &{} 0.2232 &{} 0.3629 \\ 0.9012 &{} 0.3629 &{} 1 &{} 0.4231 &{} 0.09632 &{} 0.2232 &{} 0.6104 \\ 0.4231 &{} 0.3629 &{} 0.4231 &{} 1 &{} 0.09632 &{} 0.2232 &{} 0.4231 \\ 0.09632 &{} 0.09632 &{} 0.09632 &{} 0.09632 &{} 1 &{} 0.09632 &{} 0.09632 \\ 0.2232 &{} 0.2232 &{} 0.2232 &{} 0.2232 &{} 0.09632 &{} 1 &{} 0.2232 \\ 0.6105 &{} 0.3629 &{} 0.6105 &{} 0.4231 &{} 0.09632 &{} 0.2232 &{} 1 \\ \end{array} \right) . \end{aligned}$$

Step 6: In the light of Eq. (19) to generate a $\zeta$-cutting matrix $C_{\zeta }=\left( \zeta \rho _{ij} \right) _{7 \times 7}$ from which all plausible classes of the softwares ${\mathcal {O}}_i(i = 1,2,..., 7)$ can be derived:

(i) If $0\le \zeta \le 0.09632$, then ${\mathcal {O}}_i(i = 1,2,..., 7)$ are of the same type:
  $$\begin{aligned} \left\{ {\mathcal {O}}_1,{\mathcal {O}}_2,{\mathcal {O}}_3,{\mathcal {O}}_4,{\mathcal {O}}_5, {\mathcal {O}}_6,{\mathcal {O}}_7 \right\} . \end{aligned}$$
(ii) If $0.09632< \zeta \le 0.2232$, then ${\mathcal {O}}_i(i = 1,2,..., 7)$ are categorized into two types:
  $$\begin{aligned} \left\{ {\mathcal {O}}_1,{\mathcal {O}}_2,{\mathcal {O}}_3,{\mathcal {O}}_4,{\mathcal {O}}_6,{\mathcal {O}}_7 \right\} ,\left\{ {\mathcal {O}}_5\right\} . \end{aligned}$$
(iii) If $0.2232< \zeta \le 0.3629$, then ${\mathcal {O}}_i(i = 1,2,..., 7)$ are categorized into three types:
  $$\begin{aligned} \left\{ {\mathcal {O}}_1,{\mathcal {O}}_2,{\mathcal {O}}_3,{\mathcal {O}}_4,{\mathcal {O}}_7 \right\} ,\left\{ {\mathcal {O}}_5\right\} ,\left\{ {\mathcal {O}}_6\right\} . \end{aligned}$$
(iv) If $0.3629< \zeta \le 0.4231$, then ${\mathcal {O}}_i(i = 1,2,..., 7)$ are categorized into four types:
  $$\begin{aligned} \left\{ {\mathcal {O}}_1,{\mathcal {O}}_3,{\mathcal {O}}_4,{\mathcal {O}}_7\right\} ,\left\{ {\mathcal {O}}_2\right\} , \left\{ {\mathcal {O}}_5 \right\} ,\left\{ {\mathcal {O}}_6\right\} . \end{aligned}$$
(v) If $0.4231< \zeta \le 0.6104$, then ${\mathcal {O}}_i(i = 1,2,..., 7)$ are categorized into five types:
  $$\begin{aligned} \left\{ {\mathcal {O}}_1,{\mathcal {O}}_3,{\mathcal {O}}_7\right\} ,\left\{ {\mathcal {O}}_2\right\} , \left\{ {\mathcal {O}}_4 \right\} ,\left\{ {\mathcal {O}}_5\right\} ,\left\{ {\mathcal {O}}_6\right\} . \end{aligned}$$
(vi) If $0.6104< \zeta \le 0.6105$, then ${\mathcal {O}}_i(i = 1,2,..., 7)$ are categorized into six types:
  $$\begin{aligned} \left\{ {\mathcal {O}}_1,{\mathcal {O}}_3\right\} ,\left\{ {\mathcal {O}}_2\right\} , \left\{ {\mathcal {O}}_4 \right\} ,\left\{ {\mathcal {O}}_5\right\} ,\left\{ {\mathcal {O}}_6\right\} ,\left\{ {\mathcal {O}}_7\right\} . \end{aligned}$$
(vii) If $0.6105< \zeta \le 1$, then ${\mathcal {O}}_i(i = 1,2,..., 7)$ are categorized into seven types:
  $$\begin{aligned} \left\{ {\mathcal {O}}_1\right\} ,\left\{ {\mathcal {O}}_2\right\} , \left\{ {\mathcal {O}}_3\right\} ,\left\{ {\mathcal {O}}_4 \right\} ,\left\{ {\mathcal {O}}_5\right\} ,\left\{ {\mathcal {O}}_6\right\} ,\left\{ {\mathcal {O}}_7\right\} . \end{aligned}$$

From Case 1 through Case 6, we can notice that the corresponding correlation matrix is $C^4$ in each case, requiring three iterations in each instance. Therefore, the framed method is stable from the aspect of the number of iterations, since changing the value of q has no impact on its iterations.

From the above experimental results, we can further find that the softwares ${\mathcal {O}}_i(i = 1,2,..., 7)$ are categorised into a single class in Cases 1, 2,..., 6 if $\zeta \le 0.6220$, $\zeta \le 0.5425$, $\zeta \le 0.4026$, $\zeta \le 0.2425$, $\zeta \le 0.1623$,$\zeta \le 0.09632$, respectively. Secondly, these softwares categorize into two classes in Case 1, 2,....,6 if $\zeta \le 0.7030$, $\zeta \le 0.5862$,$\zeta \le 0.4693$, $\zeta \le 0.4015$,$\zeta \le 0.3308$, $\zeta \le 0.2232$, respectively. Similarly, the bound of $\zeta$ can be seen for the next four scenarios. These results indicate that increase in the value of *q* decrease the upper bound of $\zeta$ for classifications.

What’s more, in Cases 1 and 2 (two categorization scenario), we find that ${\mathcal {O}}_2$ belongs to a separate class than the other softwares. While Cases 3-6 classify ${\mathcal {O}}_5$ as a distinct category from the others. Next, in the six categorization scenario, the results of Cases 4 and 6 do not match those of Cases 1, 2, 3, and 5. There are significant variances across the remaining situations. Therefore, the framed method is extremely sensitive to ‘q’ from a classification point.

#### How to choose the optimal value of q ?

To obtain a reasonable value for the parameter *q*, it must be determined based on the evaluation values provided by the DMs, and it may pick the lowest integer q meeting the inequality ${\mu }^q+{\nu }^q\le 1$. For instance, if the evaluating value provided by the DM is $\left\langle 0.8, 0.7\right\rangle$, we may set the parameter *q* to 3 since $0.8^2 + 0.7^2 > 1$ and $0.8^3 + 0.7^3< 1$, where $q=3$ is the smallest integer. If the DM wants to make a judgment based on complicated data, just increase q to enlarge the information representation space of q-ROPFLSs.

### Criteria weight analysis

The present part performs sensitivity analysis by varying several criteria weights to ensure the robustness of the created approach. To accomplish this, we switch the weights of any two criteria while maintaining the weights of the other criteria constant. In the case of four criteria, there are six possible cases: ${\mathcal {C}}_1-{\mathcal {C}}_4$, ${\mathcal {C}}_1-{\mathcal {C}}_3$, ${\mathcal {C}}_1-{\mathcal {C}}_2$, ${\mathcal {C}}_2-{\mathcal {C}}_4$, ${\mathcal {C}}_2-{\mathcal {C}}_3$, ${\mathcal {C}}_3-{\mathcal {C}}_4$.

**Case 1:** Interchanging ${\mathcal {C}}_1$ and ${\mathcal {C}}_4$:

Utilizing Eq. (14) the resulting correlation matrix is obtained as:

$$\begin{aligned} C=\left( \begin{array}{ccccccc} 1 &{} 0.1324 &{} 0.7194 &{} 0.4863 &{} 0.5121 &{} 0.7074 &{} 0.6604 \\ 0.1324 &{} 1&{} 0.5883 &{} 0.3970 &{} 0.4839 &{} 0.3424 &{} 0.4401 \\ 0.7194 &{} 0.5883 &{} 1&{} 0.8337 &{} 0.6858 &{} 0.7019 &{} 0.8002 \\ 0.5339 &{} 0.3970 &{} 0.8337 &{} 1 &{} 0.7763 &{} 0.6429 &{} 0.4393 \\ 0.5121 &{} 0.4839 &{} 0.6858 &{} 0.7763 &{} 1 &{} 0.4656 &{} 0.3072 \\ 0.7074 &{} 0.3424 &{} 0.7019 &{} 0.6429 &{} 0.4656 &{} 1 &{} 0.7602 \\ 0.6604 &{} 0.4276 &{} 0.8759 &{} 0.4393 &{} 0.3072 &{} 0.7602 &{} 1 \\ \end{array} \right) . \end{aligned}$$

Step 5: Find out the equivalent correlation matrix:

$$\begin{aligned}{} & {} C^2=C\circ C=\left( \begin{array}{ccccccc} 1 &{} 0.5883 &{} 0.7194 &{} 0.7194 &{} 0.7194 &{} 0.7019 &{} 0.7194 \\ 0.5883 &{} 1 &{} 0.5883 &{} 0.5883 &{} 0.5883 &{} 0.5883 &{} 0.5883 \\ 0.7194 &{} 0.5883 &{} 1 &{} 0.8337 &{} 0.7763 &{} 0.7602 &{} 0.8002 \\ 0.7194 &{} 0.8337 &{} 0.8337 &{} 1 &{} 0.7763 &{} 0.7019 &{} 0.8002 \\ 0.6858 &{} 0.5883 &{} 0.7763 &{} 0.7763 &{} 1 &{} 0.6858 &{} 0.6858 \\ 0.7074 &{} 0.5883 &{} 0.7602 &{} 0.7074 &{} 0.6858 &{} 1 &{} 0.7602 \\ 0.7194 &{} 0.5883 &{} 0.8759 &{} 0.8337 &{} 0.6858 &{} 0.7602 &{} 1 \\ \end{array} \right) \\{} & {} \quad C^4=C^2\circ C^2=\left( \begin{array}{ccccccc} 1 &{} 0.7194 &{} 0.7194 &{} 0.7194 &{} 0.7194 &{} 0.7194 &{} 0.7194 \\ 0.5883 &{} 1 &{} 0.5883 &{} 0.5883 &{} 0.5883 &{} 0.5883 &{} 0.5883 \\ 0.7194 &{} 0.8337 &{} 1 &{} 0.8337 &{} 0.7763 &{} 0.7602 &{} 0.8002 \\ 0.7194 &{} 0.8337 &{} 0.8337 &{} 1 &{} 0.7763 &{} 0.7602 &{} 0.8002 \\ 0.7194 &{} 0.7763 &{} 0.7763 &{} 0.7763 &{} 1 &{} 0.7602 &{} 0.7763 \\ 0.7194 &{} 0.7074 &{} 0.7602 &{} 0.7602 &{} 0.7602 &{} 1 &{} 0.7602 \\ 0.7194 &{} 0.8337 &{} 0.8759 &{} 0.8337 &{} 0.7763 &{} 0.7602 &{} 1 \\ \end{array} \right) \\{} & {} \quad C^8=C^4\circ C^4=\left( \begin{array}{ccccccc} 1 &{} 0.7194 &{} 0.7194 &{} 0.7194 &{} 0.7194 &{} 0.7194 &{} 0.7194 \\ 0.5883 &{} 1 &{} 0.5883 &{} 0.5883 &{} 0.5883 &{} 0.5883 &{} 0.5883 \\ 0.7194 &{} 0.8337 &{} 1 &{} 0.8337 &{} 0.7763 &{} 0.7602 &{} 0.8002 \\ 0.7194 &{} 0.8337 &{} 0.8337 &{} 1 &{} 0.7763 &{} 0.7602 &{} 0.8002 \\ 0.7194 &{} 0.7763 &{} 0.7763 &{} 0.7763 &{} 1 &{} 0.7602 &{} 0.7763 \\ 0.7194 &{} 0.7074 &{} 0.7602 &{} 0.7602 &{} 0.7602 &{} 1 &{} 0.7602 \\ 0.7194 &{} 0.8337 &{} 0.8759 &{} 0.8337 &{} 0.7763 &{} 0.7602 &{} 1 \\ \end{array} \right) . \end{aligned}$$

Step 6: In the light of Eq. (19) to generate a $\zeta$-cutting matrix $C_{\zeta }=\left( \zeta \rho _{ij} \right) _{7 \times 7}$ from which all plausible classes of the softwares ${\mathcal {O}}_i(i = 1,2,..., 7)$ can be derived:

(i) If $0\le \zeta \le 0.5889$, then ${\mathcal {O}}_i(i = 1,2,..., 7)$ are of the same type:
  $$\begin{aligned} \left\{ {\mathcal {O}}_1,{\mathcal {O}}_2,{\mathcal {O}}_3,{\mathcal {O}}_4,{\mathcal {O}}_5, {\mathcal {O}}_6,{\mathcal {O}}_7 \right\} . \end{aligned}$$
(ii) If $0.5889< \zeta \le 0.7074$, then ${\mathcal {O}}_i(i = 1,2,..., 7)$ are categorized into two types:
  $$\begin{aligned} \left\{ {\mathcal {O}}_1,{\mathcal {O}}_3,{\mathcal {O}}_4,{\mathcal {O}}_5,{\mathcal {O}}_6,{\mathcal {O}}_7 \right\} ,\left\{ {\mathcal {O}}_2\right\} . \end{aligned}$$
(iii) If $0.7074< \zeta \le 0.7193$, then ${\mathcal {O}}_i(i = 1,2,..., 7)$ are categorized into three types:
  $$\begin{aligned} \left\{ {\mathcal {O}}_1,{\mathcal {O}}_3,{\mathcal {O}}_4,{\mathcal {O}}_5,{\mathcal {O}}_7 \right\} ,\left\{ {\mathcal {O}}_2\right\} ,\left\{ {\mathcal {O}}_6\right\} . \end{aligned}$$
(iv) If $0.7193< \zeta \le 0.7602$, then ${\mathcal {O}}_i(i = 1,2,..., 7)$ are categorized into four types:
  $$\begin{aligned} \left\{ {\mathcal {O}}_3,{\mathcal {O}}_4,{\mathcal {O}}_5,{\mathcal {O}}_7\right\} ,\left\{ {\mathcal {O}}_1\right\} , \left\{ {\mathcal {O}}_2 \right\} ,\left\{ {\mathcal {O}}_6\right\} . \end{aligned}$$
(v) If $0.77602< \zeta \le 0.7763$, then ${\mathcal {O}}_i(i = 1,2,..., 7)$ are categorized into five types:
  $$\begin{aligned} \left\{ {\mathcal {O}}_1\right\} , \left\{ {\mathcal {O}}_2 \right\} ,\left\{ {\mathcal {O}}_3,{\mathcal {O}}_4,{\mathcal {O}}_5\right\} ,\left\{ {\mathcal {O}}_6\right\} ,\left\{ {\mathcal {O}}_7\right\} . \end{aligned}$$
(vi) If $0.7763< \zeta \le 0.8337$, then ${\mathcal {O}}_i(i = 1,2,..., 7)$ are categorized into six types:
  $$\begin{aligned} \left\{ {\mathcal {O}}_1\right\} , \left\{ {\mathcal {O}}_2 \right\} ,\left\{ {\mathcal {O}}_3,{\mathcal {O}}_4\right\} ,\left\{ {\mathcal {O}}_5\right\} ,\left\{ {\mathcal {O}}_6\right\} ,\left\{ {\mathcal {O}}_7\right\} . \end{aligned}$$
(vii) If $0.8337< \zeta \le 1$, then ${\mathcal {O}}_i(i = 1,2,..., 7)$ are categorized into seven types:
  $$\begin{aligned} \left\{ {\mathcal {O}}_1\right\} ,\left\{ {\mathcal {O}}_2\right\} , \left\{ {\mathcal {O}}_3\right\} ,\left\{ {\mathcal {O}}_4 \right\} ,\left\{ {\mathcal {O}}_5\right\} ,\left\{ {\mathcal {O}}_6\right\} ,\left\{ {\mathcal {O}}_7\right\} . \end{aligned}$$

**Case 2:** Interchanging ${\mathcal {C}}_1$ and ${\mathcal {C}}_3$:

Step 4: Utilizing Eq. (14) the resulting correlation matrix is obtained as:

$$\begin{aligned} C=\left( \begin{array}{ccccccc} 1 &{} 0.1291 &{} 0.7083 &{} 0.4478 &{} 0.4414 &{} 0.6697 &{} 0.6692 \\ 0.1291 &{} 1&{} 0.6381 &{} 0.4601 &{} 0.5789 &{} 0.3350 &{} 0.4213 \\ 0.7083 &{} 0.6381 &{} 1&{} 0.8074 &{} 0.6791 &{} 0.6873 &{} 0.8089 \\ 0.4952 &{} 0.4601 &{} 0.8074 &{} 1 &{} 0.7502 &{} 0.6680 &{} 0.4463 \\ 0.4414 &{} 0.5789 &{} 0.6791 &{} 0.7502 &{} 1 &{} 0.4234 &{} 0.2932 \\ 0.6697 &{} 0.3350 &{} 0.6873 &{} 0.6680 &{} 0.4234 &{} 1 &{} 0.7553 \\ 0.6692 &{} 0.4066 &{} 0.8606 &{} 0.4463 &{} 0.2932 &{} 0.7553 &{} 1 \\ \end{array} \right) . \end{aligned}$$

Step 5: Find out the equivalent correlation matrix:

$$\begin{aligned}{} & {} C^2=C\circ C=\left( \begin{array}{ccccccc} 1 &{} 0.6381 &{} 0.7083 &{} 0.7083 &{} 0.6791 &{} 0.6873 &{} 0.7083 \\ 0.6381 &{} 1 &{} 0.6381 &{} 0.6381 &{} 0.6381 &{} 0.6381 &{} 0.6381 \\ 0.7083 &{} 0.6381 &{} 1 &{} 0.8074 &{} 0.7502 &{} 0.7553 &{} 0.8089 \\ 0.7083 &{} 0.6381 &{} 0.8074 &{} 1 &{} 0.7502 &{} 0.6873 &{} 0.8074 \\ 0.6791 &{} 0.6381 &{} 0.7502 &{} 0.7502 &{} 1 &{} 0.6791 &{} 0.6791 \\ 0.6873 &{} 0.6381 &{} 0.7553 &{} 0.6873&{} 0.6791 &{} 1 &{} 0.7553 \\ 0.7083 &{} 0.6381 &{} 0.8606 &{} 0.8074 &{} 0.6791 &{} 0.7553 &{} 1 \\ \end{array} \right) \\{} & {} \quad C^4=C^2\circ C^2=\left( \begin{array}{ccccccc} 1 &{} 0.6381 &{} 0.7083 &{} 0.7083 &{} 0.7083 &{} 0.7083 &{} 0.7083 \\ 0.6381 &{} 1 &{} 0.6381 &{} 0.6381 &{} 0.6381 &{} 0.6381 &{} 0.6381 \\ 0.7083 &{} 0.6381 &{} 1 &{} 0.8074 &{} 0.7502 &{} 0.7553 &{} 0.8089 \\ 0.7083 &{} 0.6381 &{} 0.8074 &{} 1 &{} 0.7502 &{} 0.7553 &{} 0.8074 \\ 0.7083 &{} 0.6381 &{} 0.7502 &{} 0.7502 &{} 1 &{} 0.7502 &{} 0.7502 \\ 0.7083 &{} 0.6381 &{} 0.7553 &{} 0.7553 &{} 0.7502 &{} 1 &{} 0.7553 \\ 0.7083 &{} 0.6381 &{} 0.8606 &{} 0.8074 &{} 0.7502 &{} 0.7553 &{} 1 \\ \end{array} \right) \\{} & {} \quad C^8=C^4\circ C^4=\left( \begin{array}{ccccccc} 1 &{} 0.6381 &{} 0.7083 &{} 0.7083 &{} 0.7083 &{} 0.7083 &{} 0.7083 \\ 0.6381 &{} 1 &{} 0.6381 &{} 0.6381 &{} 0.6381 &{} 0.6381 &{} 0.6381 \\ 0.7083 &{} 0.6381 &{} 1 &{} 0.8074 &{} 0.7502 &{} 0.7553 &{} 0.8089 \\ 0.7083 &{} 0.6381 &{} 0.8074 &{} 1 &{} 0.7502 &{} 0.7553 &{} 0.8074 \\ 0.7083 &{} 0.6381 &{} 0.7502 &{} 0.7502 &{} 1 &{} 0.7502 &{} 0.7502 \\ 0.7083 &{} 0.6381 &{} 0.7553 &{} 0.7553 &{} 0.7502 &{} 1 &{} 0.7553 \\ 0.7083 &{} 0.6381 &{} 0.8606 &{} 0.8074 &{} 0.7502 &{} 0.7553 &{} 1 \\ \end{array} \right) . \end{aligned}$$

Step 6: In the light of Eq. (19) to generate a $\zeta$-cutting matrix $C_{\zeta }=\left( \zeta \rho _{ij} \right) _{7 \times 7}$ from which all plausible classes of the softwares ${\mathcal {O}}_i(i = 1,2,..., 7)$ can be derived:

(i) If $0\le \zeta \le 0.6381$, then ${\mathcal {O}}_i(i = 1,2,..., 7)$ are of the same type:
  $$\begin{aligned} \left\{ {\mathcal {O}}_1,{\mathcal {O}}_2,{\mathcal {O}}_3,{\mathcal {O}}_4,{\mathcal {O}}_5, {\mathcal {O}}_6,{\mathcal {O}}_7 \right\} . \end{aligned}$$
(ii) If $0.6381< \zeta \le 0.7083$, then ${\mathcal {O}}_i(i = 1,2,..., 7)$ are categorized into two types:
  $$\begin{aligned} \left\{ {\mathcal {O}}_1,{\mathcal {O}}_3,{\mathcal {O}}_4,{\mathcal {O}}_5,{\mathcal {O}}_6,{\mathcal {O}}_7 \right\} ,\left\{ {\mathcal {O}}_2\right\} . \end{aligned}$$
(iii) If $0.7083< \zeta \le 0.7502$, then ${\mathcal {O}}_i(i = 1,2,..., 7)$ are categorized into three types:
  $$\begin{aligned} \left\{ {\mathcal {O}}_1\right\} ,\left\{ {\mathcal {O}}_2\right\} ,\left\{ {\mathcal {O}}_3,{\mathcal {O}}_4,{\mathcal {O}}_5,{\mathcal {O}}_6,{\mathcal {O}}_7 \right\} . \end{aligned}$$
(iv) If $0.7502< \zeta \le 0.7553$, then ${\mathcal {O}}_i(i = 1,2,..., 7)$ are categorized into four types:
  $$\begin{aligned} \left\{ {\mathcal {O}}_1\right\} , \left\{ {\mathcal {O}}_2\right\} ,\left\{ {\mathcal {O}}_3,{\mathcal {O}}_4\right\} , \left\{ {\mathcal {O}}_5 \right\} ,\left\{ {\mathcal {O}}_6,{\mathcal {O}}_7\right\} . \end{aligned}$$
(v) If $0.7553< \zeta \le 0.8074$, then ${\mathcal {O}}_i(i = 1,2,..., 7)$ are categorized into five types:
  $$\begin{aligned} \left\{ {\mathcal {O}}_1\right\} , \left\{ {\mathcal {O}}_2\right\} ,\left\{ {\mathcal {O}}_3,{\mathcal {O}}_4,{\mathcal {O}}_7\right\} ,\left\{ {\mathcal {O}}_5\right\} , \left\{ {\mathcal {O}}_6 \right\} . \end{aligned}$$
(vi) If $0.8074< \zeta \le 0.8089$, then ${\mathcal {O}}_i(i = 1,2,..., 7)$ are categorized into six types:
  $$\begin{aligned} \left\{ {\mathcal {O}}_1\right\} ,\left\{ {\mathcal {O}}_2\right\} , \left\{ {\mathcal {O}}_4 \right\} ,\left\{ {\mathcal {O}}_5\right\} ,\left\{ {\mathcal {O}}_6\right\} ,\left\{ {\mathcal {O}}_3,{\mathcal {O}}_7\right\} . \end{aligned}$$
(vii) If $0.8089< \zeta \le 1$, then ${\mathcal {O}}_i(i = 1,2,..., 7)$ are categorized into seven types:
  $$\begin{aligned} \left\{ {\mathcal {O}}_1\right\} ,\left\{ {\mathcal {O}}_2\right\} , \left\{ {\mathcal {O}}_3\right\} ,\left\{ {\mathcal {O}}_4 \right\} ,\left\{ {\mathcal {O}}_5\right\} ,\left\{ {\mathcal {O}}_6\right\} ,\left\{ {\mathcal {O}}_7\right\} . \end{aligned}$$

Similarly, the proposed approach can be used to solve the other cases. In Cases 3, 4, 5, and 6, we will find that the software is of the same type for $0< \zeta \le 0.6121$, $0< \zeta \le 0.6145$, $0< \zeta \le 0.6301$, and $0< \zeta \le 0.6031$, respectively. It is evident from this that the upper bounds of the intervals are quite close to each other, indicating that the introduced algorithm is stable with respect to criteria weight fluctuations.

### Comparative Illustration

This section compares the devised clustering algorithm with previous methods, including intuitionistic fuzzy and q-rung orthopair fuzzy clustering algorithms^36,40^.

Consider the Jiang et al. clustering of four construction materials problem^41^. Let $\left\{ {\mathcal {O}}_1,{\mathcal {O}}_2,{\mathcal {O}}_3,{\mathcal {O}}_4 \right\}$ represent the four types of construction materials: sealant, floor varnish, wall paint, and carpet. Assume each of these materials has four criteria ${\mathcal {C}}_1$, ${\mathcal {C}}_2$, ${\mathcal {C}}_3$, and ${\mathcal {C}}_4$ with the weight vector $\varpi =\left( 0.2,0.2,0.3,0.3\right)$. DMs give their views based on the LTS, *S* (as taken previously).

The data collected for the four materials by three professionals according to each criterion is recorded in Tables 5, 6 and 7.

**Table 5:** q-ROPFL data provided by ${\mathcal {D}}_1$.

	${\mathcal {C}}_1$	${\mathcal {C}}_2$	${\mathcal {C}}_3$	${\mathcal {C}}_4$
${\mathcal {O}}_1$	$\left( \left( s_{2},0\right) ,\left\langle 0.25, 0.40\right\rangle \right)$	$\left( \left( s_{5},0\right) ,\left\langle 0.50, 0.35\right\rangle \right)$	$\left( \left( s_{7},0\right) ,\left\langle 0.45, 0.20\right\rangle \right)$	$\left( \left( s_{6},0\right) ,\left\langle 0.55, 0.40\right\rangle \right)$
${\mathcal {O}}_2$	$\left( \left( s_{3},0\right) ,\left\langle 0.35, 0.20\right\rangle \right)$	$\left( \left( s_{4},0\right) ,\left\langle 0.60, 0.40\right\rangle \right)$	$\left( \left( s_{6},0\right) ,\left\langle 0.30, 0.65\right\rangle \right)$	$\left( \left( s_{2},0\right) ,\left\langle 0.40, 0.20\right\rangle \right)$
${\mathcal {O}}_3$	$\left( \left( s_{6},0\right) ,\left\langle 0.45, 0.15\right\rangle \right)$	$\left( \left( s_{4},0\right) ,\left\langle 0.25, 0.40\right\rangle \right)$	$\left( \left( s_{3},0\right) ,\left\langle 0.30, 0.30\right\rangle \right)$	$\left( \left( s_{2},0\right) ,\left\langle 0.40, 0.60\right\rangle \right)$
${\mathcal {O}}_4$	$\left( \left( s_{4},0\right) ,\left\langle 0.60, 0.30\right\rangle \right)$	$\left( \left( s_{4},0\right) ,\left\langle 0.35, 0.45\right\rangle \right)$	$\left( \left( s_{3},0\right) ,\left\langle 0.65, 0.20\right\rangle \right)$	$\left( \left( s_{6},0\right) ,\left\langle 0.40, 0.15\right\rangle \right)$

**Table 6:** q-ROPFL data provided by ${\mathcal {D}}_2$.

	${\mathcal {C}}_1$	${\mathcal {C}}_2$	${\mathcal {C}}_3$	${\mathcal {C}}_4$
${\mathcal {O}}_1$	$\left( \left( s_{3},0\right) ,\left\langle 0.50, 0.40\right\rangle \right)$	$\left( \left( s_{3},0\right) ,\left\langle 0.45, 0.35\right\rangle \right)$	$\left( \left( s_{5},0\right) ,\left\langle 0.45, 0.45\right\rangle \right)$	$\left( \left( s_{4},0\right) ,\left\langle 0.65, 0.35\right\rangle \right)$
${\mathcal {O}}_2$	$\left( \left( s_{2},0\right) ,\left\langle 0.20, 0.35\right\rangle \right)$	$\left( \left( s_{2},0\right) ,\left\langle 0.55, 0.40\right\rangle \right)$	$\left( \left( s_{6},0\right) ,\left\langle 0.35, 0.30\right\rangle \right)$	$\left( \left( s_{4},0\right) ,\left\langle 0.40, 0.40\right\rangle \right)$
${\mathcal {O}}_3$	$\left( \left( s_{1},0\right) ,\left\langle 0.70, 0.30\right\rangle \right)$	$\left( \left( s_{5},0\right) ,\left\langle 0.55, 0.40\right\rangle \right)$	$\left( \left( s_{4},0\right) ,\left\langle 0.30, 0.60\right\rangle \right)$	$\left( \left( s_{2},0\right) ,\left\langle 0.25, 0.15\right\rangle \right)$
${\mathcal {O}}_4$	$\left( \left( s_{3},0\right) ,\left\langle 0.35, 0.35\right\rangle \right)$	$\left( \left( s_{2},0\right) ,\left\langle 0.55, 0.30\right\rangle \right)$	$\left( \left( s_{5},0\right) ,\left\langle 0.40, 0.55\right\rangle \right)$	$\left( \left( s_{6},0\right) ,\left\langle 0.35, 0.45\right\rangle \right)$

**Table 7:** q-ROPFL data provided by ${\mathcal {D}}_3$.

	${\mathcal {C}}_1$	${\mathcal {C}}_2$	${\mathcal {C}}_3$	${\mathcal {C}}_4$
${\mathcal {O}}_1$	$\left( \left( s_{2},0\right) ,\left\langle 0.65, 0.30\right\rangle \right)$	$\left( \left( s_{4},0\right) ,\left\langle 0.55, 0.35\right\rangle \right)$	$\left( \left( s_{4},0\right) ,\left\langle 0.55, 0.45\right\rangle \right)$	$\left( \left( s_{5},0\right) ,\left\langle 0.30, 0.40\right\rangle \right)$
${\mathcal {O}}_2$	$\left( \left( s_{1},0\right) ,\left\langle 0.60, 0.35\right\rangle \right)$	$\left( \left( s_{3},0\right) ,\left\langle 0.45, 0.45\right\rangle \right)$	$\left( \left( s_{4},0\right) ,\left\langle 0.25, 0.50\right\rangle \right)$	$\left( \left( s_{6},0\right) ,\left\langle 0.20, 0.70\right\rangle \right)$
${\mathcal {O}}_3$	$\left( \left( s_{2},0\right) ,\left\langle 0.50, 0.50\right\rangle \right)$	$\left( \left( s_{4},0\right) ,\left\langle 0.35, 0.40\right\rangle \right)$	$\left( \left( s_{2},0\right) ,\left\langle 0.25, 0.50\right\rangle \right)$	$\left( \left( s_{5},0\right) ,\left\langle 0.50, 0.50\right\rangle \right)$
${\mathcal {O}}_4$	$\left( \left( s_{3},0\right) ,\left\langle 0.70, 0.20\right\rangle \right)$	$\left( \left( s_{7},0\right) ,\left\langle 0.45, 0.30\right\rangle \right)$	$\left( \left( s_{6},0\right) ,\left\langle 0.65, 0.30\right\rangle \right)$	$\left( \left( s_{5},0\right) ,\left\langle 0.55, 0.25\right\rangle \right)$

Here, we cluster the customers based on the appropriate degree of confidence. Note that in a clustering method, a broad range of confidence level results in the formation of viable clusters. We first employ the proposed algorithm for various values of parameter $\zeta$ as follows:

Step 1: The q-ROPFL decision matrices $M^{k}_{4 \times 4} (k = 1,2,3)$ are depicted in Tables 5-7.

Step 2: Employ the q-ROPFLA operator Eq. (6) (taking $q=1$) to aggregate all the matrices into collective decision matrix (see Table 8).

**Table 8:** Aggregated q-rung orthopair fuzzy 2-tuple linguistic decision matrix.

	${\mathcal {C}}_1$	${\mathcal {C}}_2$	${\mathcal {C}}_3$	${\mathcal {C}}_4$
${\mathcal {O}}_1$	$\left( \left( s_{2},0.3333\right) ,\left\langle 0.4918, 0.3634\right\rangle \right)$	$\left( \left( s_{4},0.0000\right) ,\left\langle 0.5017, 0.3500\right\rangle \right)$	$\left( \left( s_{5},0.3333\right) ,\left\langle 0.4856, 0.3434\right\rangle \right)$	$\left( \left( s_{5},0.0000\right) ,\left\langle 0.5205, 0.3826\right\rangle \right)$
${\mathcal {O}}_2$	$\left( \left( s_{2},0.0000\right) ,\left\langle 0.4075, 0.2904\right\rangle \right)$	$\left( \left( s_{3},0.0000\right) ,\left\langle 0.5374, 0.4160\right\rangle \right)$	$\left( \left( s_{5},0.3333\right) ,\left\langle 0.3012, 0.4603\right\rangle \right)$	$\left( \left( s_{4},0.0000\right) ,\left\langle 0.3396, 0.3826\right\rangle \right)$
${\mathcal {O}}_3$	$\left( \left( s_{3},0.0000\right) ,\left\langle 0.5647, 0.5647\right\rangle \right)$	$\left( \left( s_{4},0.3333\right) ,\left\langle 0.3969, 0.4000\right\rangle \right)$	$\left( \left( s_{3},0.0000\right) ,\left\langle 0.2837, 0.4481\right\rangle \right)$	$\left( \left( s_{3},0.0000\right) ,\left\langle 0.3918, 0.3557\right\rangle \right)$
${\mathcal {O}}_4$	$\left( \left( s_{3},0.3333\right) ,\left\langle 0.5727, 0.2759\right\rangle \right)$	$\left( \left( s_{4},0.3333\right) ,\left\langle 0.4561, 0.3434\right\rangle \right)$	$\left( \left( s_{4},-0.3333\right) ,\left\langle 0.5811, 0.3208\right\rangle \right)$	$\left( \left( s_{5},-0.3333\right) ,\left\langle 0.4401, 0.2565\right\rangle \right)$

Step 3: Since the weight vector is given by DMs, so we omit this step.

Step 4: The weighted correlation matrix of the q-ROPFLSs ${\mathcal {O}}_i(i=1,2,...,4)$ with respect to their given weight is computed as follows:

$$\begin{aligned} C=\left( \begin{array}{cccc} 1 &{} 0.9468 &{} 0.8745 &{} 0.9712 \\ 0.9468 &{} 1 &{} 0.8837 &{} 0.9017 \\ 0.8745 &{} 0.8837 &{} 1 &{} 0.9215 \\ 0.9712 &{} 0.9017 &{} 0.9215 &{} 1 \\ \end{array} \right) \end{aligned}$$

Step 5: Determine the equivalent correlation matrix:

$$\begin{aligned}{} & {} C^2=C\circ C=\left( \begin{array}{cccc} 1 &{} 0.9468 &{} 0.9215 &{} 0.9712 \\ 0.9468 &{} 1 &{} 0.9017 &{} 0.9468 \\ 0.9215 &{} 0.9017 &{} 1 &{} 0.9215 \\ 0.9712 &{} 0.9468 &{} 0.9215 &{} 1 \\ \end{array} \right) \\{} & {} \quad C^4=C^2\circ C^2=\left( \begin{array}{cccc} 1 &{} 0.9468 &{} 0.9215 &{} 0.9712 \\ 0.9468 &{} 1 &{} 0.9215 &{} 0.9468 \\ 0.9215 &{} 0.9215 &{} 1 &{} 0.9215 \\ 0.9712 &{} 0.9468 &{} 0.9215 &{} 1 \\ \end{array} \right) \\{} & {} \quad C^8=C^4\circ C^4=\left( \begin{array}{cccc} 1 &{} 0.9468 &{} 0.9215 &{} 0.9712 \\ 0.9468 &{} 1 &{} 0.9215 &{} 0.9468 \\ 0.9215 &{} 0.9215 &{} 1 &{} 0.9215 \\ 0.9712 &{} 0.9468 &{} 0.9215 &{} 1 \\ \end{array} \right) \end{aligned}$$

Step 6: In the light of Eq. (19) to generate a $\zeta$-cutting matrix $C_{\zeta }=\left( \zeta \rho _{ij} \right) _{4 \times 4}$ from which all plausible classes of the softwares ${\mathcal {O}}_i(i = 1,2,..., 4)$ can be derived:

(i) If $0\le \zeta \le 0.9215$, then ${\mathcal {O}}_i(i = 1,2,..., 4)$ are of the same type:
  $$\begin{aligned} \left\{ {\mathcal {O}}_1,{\mathcal {O}}_2,{\mathcal {O}}_3,{\mathcal {O}}_4 \right\} . \end{aligned}$$
(ii) If $0.9215< \zeta \le 0.9468$, then ${\mathcal {O}}_i(i = 1,2,..., 4)$ are categorized into two types:
  $$\begin{aligned} \left\{ {\mathcal {O}}_1,{\mathcal {O}}_2,{\mathcal {O}}_4 \right\} ,\left\{ {\mathcal {O}}_3\right\} \end{aligned}$$
(iii) If $0.9468< \zeta \le 0.9712$, then ${\mathcal {O}}_i(i = 1,2,..., 4)$ are categorized into three types:
  $$\begin{aligned} \left\{ {\mathcal {O}}_1, {\mathcal {O}}_4\right\} ,\left\{ {\mathcal {O}}_2\right\} , \left\{ {\mathcal {O}}_3\right\} \end{aligned}$$
(iv) If $0.9712< \zeta \le 1$, then ${\mathcal {O}}_i(i = 1,2,..., 4)$ are categorized into four types:
  $$\begin{aligned} \left\{ {\mathcal {O}}_1\right\} ,\left\{ {\mathcal {O}}_2\right\} , \left\{ {\mathcal {O}}_3\right\} , \left\{ {\mathcal {O}}_4\right\} \end{aligned}$$

Example 2 data can not be modeled by applying intuitionistic and q-rung orthopair fuzzy correlations^36,40^. To make the Table 8 legitimate for the implication of^40^ and^36^, we exclude the 2-tuple linguistic data from Table 8. By doing so, the resultant data are reduced to a q-rung orthopair fuzzy context (q=1) and are displayed in Table 9.

**Table 9:** Aggregated q-rung orthopair fuzzy decision matrix.

	${\mathcal {C}}_1$	${\mathcal {C}}_2$	${\mathcal {C}}_3$	${\mathcal {C}}_4$
${\mathcal {O}}_1$	$\left\langle 0.4918, 0.3634\right\rangle$	$\left\langle 0.5017, 0.3500\right\rangle$	$\left\langle 0.4856, 0.3434\right\rangle$	$\left\langle 0.5205, 0.3826\right\rangle$
${\mathcal {O}}_2$	$\left\langle 0.4075, 0.2904\right\rangle$	$\left\langle 0.5374, 0.4160\right\rangle$	$\left\langle 0.3012, 0.4603\right\rangle$	$\left\langle 0.3396, 0.3826\right\rangle$
${\mathcal {O}}_3$	$\left\langle 0.5647, 0.5647\right\rangle$	$\left\langle 0.3969, 0.4000\right\rangle$	$\left\langle 0.2837, 0.4481\right\rangle$	$\left\langle 0.3918, 0.3557\right\rangle$
${\mathcal {O}}_4$	$\left\langle 0.5727, 0.2759\right\rangle$	$\left\langle 0.4561, 0.3434\right\rangle$	$\left\langle 0.5811, 0.3208\right\rangle$	$\left\langle 0.4401, 0.2565\right\rangle$

The stepwise computations are performed by adhering to the method outlined in Ref.^36^.

Step 1: The weighted correlation matrix of the q-ROFSs ${\mathcal {O}}_i(i=1,2,...,4)$ with respect to their given weight is carried out as follows:

$$\begin{aligned} C=\left( \begin{array}{cccc} 1 &{} 0.9648 &{} 0.9564 &{} 0.9831 \\ 0.9648 &{} 1 &{} 0.9650 &{} 0.9346 \\ 0.9564 &{} 0.9650 &{} 1 &{} 0.9258 \\ 0.9831 &{} 0.9346 &{} 0.9258 &{} 1 \\ \end{array} \right) \end{aligned}$$

Step 2: Formulate the equivalent correlation matrix:

$$\begin{aligned}{} & {} C^2=C\circ C=\left( \begin{array}{cccc} 1 &{} 0.9648 &{} 0.9648 &{} 0.9831 \\ 0.9648 &{} 1 &{} 0.9650 &{} 0.9648 \\ 0.9648 &{} 0.9650 &{} 1 &{} 0.9564 \\ 0.9831 &{} 0.9648 &{} 0.9564 &{} 1 \\ \end{array} \right) \\{} & {} \quad C^4=C^2\circ C^2=\left( \begin{array}{cccc} 1 &{} 0.9648 &{} 0.9648 &{} 0.9831 \\ 0.9648 &{} 1 &{} 0.9650 &{} 0.9648 \\ 0.9648 &{} 0.9650 &{} 1 &{} 0.9648 \\ 0.9831 &{} 0.9648 &{} 0.9648 &{} 1 \\ \end{array} \right) \\{} & {} \quad C^8=C^4\circ C^4=\left( \begin{array}{cccc} 1 &{} 0.9648 &{} 0.9648 &{} 0.9831 \\ 0.9648 &{} 1 &{} 0.9650 &{} 0.9648 \\ 0.9648 &{} 0.9650 &{} 1 &{} 0.9648 \\ 0.9831 &{} 0.9648 &{} 0.9648 &{} 1 \\ \end{array} \right) \end{aligned}$$

Step 3: Generate a $\zeta$-cutting matrix $C_{\zeta }=\left( \zeta \rho _{ij} \right) _{4 \times 4}$ from which all plausible classes of the softwares ${\mathcal {O}}_i(i = 1,2,..., 4)$ can be derived:

(i) If $0\le \zeta \le 0.9648$, then ${\mathcal {O}}_i(i = 1,2,..., 4)$ are of the same type:
  $$\begin{aligned} \left\{ {\mathcal {O}}_1,{\mathcal {O}}_2,{\mathcal {O}}_3,{\mathcal {O}}_4\right\} . \end{aligned}$$
(ii) If $0.9648< \zeta \le 0.9650$, then ${\mathcal {O}}_i(i = 1,2,..., 4)$ are categorized into two types:
  $$\begin{aligned} \left\{ {\mathcal {O}}_1,{\mathcal {O}}_4\right\} , \left\{ {\mathcal {O}}_2,{\mathcal {O}}_3 \right\} \end{aligned}$$
(iii) If $0.9650< \zeta \le 0.9831$, then ${\mathcal {O}}_i(i = 1,2,..., 4)$ are categorized into three types:
  $$\begin{aligned} \left\{ {\mathcal {O}}_1,{\mathcal {O}}_4\right\} ,\left\{ {\mathcal {O}}_2\right\} , \left\{ {\mathcal {O}}_3 \right\} \end{aligned}$$
(iv) If $0.9831< \zeta \le 1$, then ${\mathcal {O}}_i(i = 1,2,..., 4)$ are categorized into four types:
  $$\begin{aligned} \left\{ {\mathcal {O}}_1\right\} ,\left\{ {\mathcal {O}}_2\right\} , \left\{ {\mathcal {O}}_3\right\} , \left\{ {\mathcal {O}}_4 \right\} \end{aligned}$$

Next, the stepwise computations are carried out by following the procedures of Ref.^40^ (fixing $\alpha =0.5$).

Step 1: The correlation matrix of the IFSs ${\mathcal {O}}_i(i=1,2,...,4)$ is determined as follows:

$$\begin{aligned} C=\left( \begin{array}{cccc} 1 &{} 0.9979 &{} 0.9972 &{} 0.9989 \\ 0.9979 &{} 1 &{} 0.9975 &{} 0.9962 \\ 0.9972 &{} 0.9975 &{} 1 &{} 0.9951 \\ 0.9989 &{} 0.9962 &{} 0.9951 &{} 1 \\ \end{array} \right) \end{aligned}$$

Step 2: Frame the equivalent correlation matrix:

$$\begin{aligned}{} & {} C^2=C\circ C=\left( \begin{array}{cccc} 1 &{} 0.9979 &{} 0.9975 &{} 0.9989 \\ 0.9979 &{} 1 &{} 0.9975 &{} 0.9979 \\ 0.9975 &{} 0.9975 &{} 1 &{} 0.9972 \\ 0.9989 &{} 0.9979 &{} 0.9972 &{} 1 \\ \end{array} \right) \\{} & {} \quad C^4=C^2\circ C^2=\left( \begin{array}{cccc} 1 &{} 0.9979 &{} 0.9975 &{} 0.9989 \\ 0.9979 &{} 1 &{} 0.9975 &{} 0.9979 \\ 0.9975 &{} 0.9975 &{} 1 &{} 0.9975 \\ 0.9989 &{} 0.9979 &{} 0.9975 &{} 1 \\ \end{array} \right) \\{} & {} \quad C^8=C^4\circ C^4=\left( \begin{array}{cccc} 1 &{} 0.9979 &{} 0.9975 &{} 0.9989 \\ 0.9979 &{} 1 &{} 0.9975 &{} 0.9979 \\ 0.9975 &{} 0.9975 &{} 1 &{} 0.9975 \\ 0.9989 &{} 0.9979 &{} 0.9975 &{} 1 \\ \end{array} \right) \end{aligned}$$

Step 3: Generate a $\zeta$-cutting matrix $C_{\zeta }=\left( \zeta \rho _{ij} \right) _{7 \times 7}$ from which all plausible classes of the softwares ${\mathcal {O}}_i(i = 1,2,..., 4)$ can be derived:

(i) If $0\le \zeta \le 0.9975$, then ${\mathcal {O}}_i(i = 1,2,..., 4)$ are of the same type:
  $$\begin{aligned} \left\{ {\mathcal {O}}_1,{\mathcal {O}}_2,{\mathcal {O}}_3,{\mathcal {O}}_4 \right\} . \end{aligned}$$
(ii) If $0.9975< \zeta \le 0.9979$, then ${\mathcal {O}}_i(i = 1,2,..., 4)$ are categorized into two types:
  $$\begin{aligned} \left\{ {\mathcal {O}}_3\right\} , \left\{ {\mathcal {O}}_1,{\mathcal {O}}_2,{\mathcal {O}}_4 \right\} \end{aligned}$$
(iii) If $0.9979< \zeta \le 0.9989$, then ${\mathcal {O}}_i(i = 1,2,..., 4)$ are categorized into three types:
  $$\begin{aligned} \left\{ {\mathcal {O}}_1,{\mathcal {O}}_4\right\} ,\left\{ {\mathcal {O}}_2\right\} , \left\{ {\mathcal {O}}_3 \right\} \end{aligned}$$
(iv) If $0.9989< \zeta \le 1$, then ${\mathcal {O}}_i(i = 1,2,..., 4)$ are categorized into four types:
  $$\begin{aligned} \left\{ {\mathcal {O}}_1\right\} ,\left\{ {\mathcal {O}}_2\right\} , \left\{ {\mathcal {O}}_3\right\} ,\left\{ {\mathcal {O}}_4 \right\} \end{aligned}$$

By analyzing the results generated by the Bashir et al. approach^36^, we can find out that when the number of classes is two, the alternatives ${\mathcal {O}}_2$ and ${\mathcal {O}}_3$ are clustered into a single class, but accordingly to the results derived by the suggested and^40^ method, ${\mathcal {O}}_2$ is clustered into the class of ${\mathcal {O}}_1$ and ${\mathcal {O}}_4$. Further , we can see that our developed methodology has started classification when $\zeta = 0.9215$. However, the method of^36^ and^40^ do not start classification until $\zeta =0.9648$ and 0.9975, respectively. Our clustering results have a quicker convergence rate than^36,40^, therefore they can more accurately depict the distinction between groups. Notice that the present techniques^36,40^ can only process numeric data. Their failure to cope with linguistic arguments has resulted in significant information loss. Whereas the framed algorithm is capable for a linguistic preference structure with symbolic translation parameters of linguistic arguments of solving the MCGDM problems with completely unknown weight information.

Singh et al.^40^ has used a two-parametric correlation coefficient in his work, so expanding the range of confidence level, and by modifying the values of these two parameters, we can produce the equivalent matrix with less iterations. Despite this advantage, their model fails in the most of complicated problems due to the high constraints on its characteristic functions. For example, it cannot manage the information for the value $\left\langle 0.5,0.6 \right\rangle$, but the created approach provides an adaptable parameter that enables the easy development of this kind of data. In addition, the approach of Basir et al.^36^ is based on known weights, and the method^40^ is based on correlation coefficient rather than weighted correlation coefficient; thus pay no attention to criteria weights. This ignorance may result in some incorrect clusters.

The main benefits of the described clustering algorithm over the past ones are talked over:

(i) The proposed method is suitable for a linguistic preference structure with symbolic translation parameters of linguistic arguments that are highly effective in dealing with ambiguity in the MCGDM problem. While using q-ROPFLSs, the DMs remain easier for data collection and avoid any information loss.
(ii) Unlike the prevailing techniques^36,40^, the framed algorithm aims to ascertain the weights of the evaluation criteria.
(iii) The presented method is capable for solving the group decision making problems in the context of q-ROPTLSs. Whereas the existing methods^36,40^ work only for individual decision matrix and fail to model the multi-experts problem. Also the rate of convergence of the developed clustering model is faster than^36,40^, can be analyzed from the above comparison.

The presented structure also has disadvantages, which are outlined below:

(i) In practice, DMs have different knowledge, proficiency and experiences and therefore the importance of each DM may not be equal. In present study this difference of knowledge and experiences (relative weight) of each DM is not considered and equal weight has been assigned to each DM.
(ii) The formulated correlation coefficients are information energy-based measures, whose results lie inside the interval [0, 1], and hence cannot be used to indicate the negative correlation between two variables.

## Concluding remarks

In this article, an intriguing study based on q-ROPFL clustering algorithm employed for classification problems in decision making was presented. For this, we explored the ideas of the information energy and covariance of q-ROPFLSs, and then presented a correlation coefficient. We additionally defined the weighted covariance and correlation coefficient of the q-ROPFLSs. Also, some desired properties and results of the proposed information energy and correlation coefficients were argued. Furthermore, some theoretical development, including the notion of composition matrix, correlation matrix, and equivalent correlation matrix via the proposed correlation coefficients, was given, and then proposed an algorithm for clustering q-ROPTLSs. The presented method works well with symbolic translation parameters of linguistic arguments in a linguistic preference structure. As opposed to conventional decision-making techniques, our suggested clustering algorithm uses q-ROPFLSs, which consistently prevent any loss of information. A practical problem concerning the clustering of construction materials was addressed, and a detailed sensitivity analysis was also performed. It was noticed the parameters *q* and $\zeta$ indeed have an impact on the clustering of alternatives. Finally, a comparison example was provided, and it was found that the results of the framed algorithm have more rapid convergence, which confirms the practicality and superiority of the developed approach.

When employing the proposed clustering algorithm based on q-ROPFLSs, further work is still required. To broaden the application range of the present clustering algorithm, it needs to assign weights for different DMs^19^. Secondly, the introduced concepts can be explored for other extensions of fuzzy theory, which will develop many interesting structures, results, and applications. In addition, it is also a worthy research topic to expand the range of the devised correlation coefficients to the interval $[-1,1]$^42^.

## Data Availability

All data generated or analysed during this study are included in this published article.

## Abbreviations

q-ROPFLS: q-rung orthopair fuzzy 2-tuple linguistic set
q-ROPFL: q-rung orthopair fuzzy 2-tuple linguistic
MCGDM: Multi-Criteria Group Decision-Making
LVR: Linguistic variable
2TL: 2-Tuple linguistic
P2TLN: Pythagorean 2TL number
DMs: Decision makers
q-ROPFLN: q-rung orthopair 2-tuple linguistic number
IFSs: Intuitionistic fuzzy sets
CIM: Computer-integrated manufacturing
q-ROPFLWA: q-rung orthopair fuzzy 2-tuple linguistic weighted averaging
q-ROPFLA: q-rung orthopair fuzzy 2-tuple linguistic averaging
