# Author Correction: q-rung orthopair fuzzy 2-tuple linguistic clustering algorithm and its applications to clustering analysis

**DOI:** 10.1038/s41598-023-30738-1

**Published:** 2023-03-06

**Authors:** Fatima Abbas, Jawad Ali, Wali Khan Mashwani, Muhammad I. Syam

**Affiliations:** 1 Institute of Numerical Sciences, Gomal University, D. I. Khan, KPK Pakistan; 2 Institute of Numerical Sciences, Kohat University of Science and Technology, Kohat, KPK Pakistan; 3 Department of Mathematical Sciences, United Arab Emirates University, P. O. Box 15551, Al-Ain, UAE

Correction to: *Scientific Reports* https://doi.org/10.1038/s41598-023-29932-y, published online 16 February 2023

In the original version of this Article, Wali Khan Mashwani was incorrectly affiliated with “Department of Mathematical Sciences, United Arab Emirates University, P. O. Box 15551 Al-Ain, UAE”. The correct affiliation is listed below.

Institute of Numerical Sciences, Kohat University of Science and Technology, Kohat, KPK, Pakistan.

The original Article has been corrected.

